# Proteolytic Activity-Independent Activation of the Immune Response by Gingipains from Porphyromonas gingivalis

**DOI:** 10.1128/mbio.03787-21

**Published:** 2022-05-02

**Authors:** Izabela Ciaston, Joanna Budziaszek, Dorota Satala, Barbara Potempa, Andrew Fuchs, Maria Rapala-Kozik, Danuta Mizgalska, Ewelina Dobosz, Richard J. Lamont, Jan Potempa, Joanna Koziel

**Affiliations:** a Department of Microbiology, Faculty of Biochemistry, Biophysics and Biotechnology, Jagiellonian University, Krakow, Poland; b Department of Comparative Biochemistry and Bioanalytic, Faculty of Biochemistry, Biophysics and Biotechnology, Jagiellonian University, Krakow, Poland; c Department of Oral Immunity and Infectious Diseases, University of Louisville School of Dentistry, University of Louisville, Louisville, Kentucky, USA; Emory University School of Medicine

**Keywords:** *Porphyromonas gingivalis*, gingipains, inflammation, periodontitis, epidermal growth factor receptor (EGFR)

## Abstract

Porphyromonas gingivalis, a keystone pathogen in periodontitis (PD), produces cysteine proteases named gingipains (RgpA, RgpB, and Kgp), which strongly affect the host immune system. The range of action of gingipains is extended by their release as components of outer membrane vesicles, which efficiently diffuse into surrounding gingival tissues. However, away from the anaerobic environment of periodontal pockets, increased oxygen levels lead to oxidation of the catalytic cysteine residues of gingipains, inactivating their proteolytic activity. In this context, the influence of catalytically inactive gingipains on periodontal tissues is of significant interest. Here, we show that proteolytically inactive RgpA induced a proinflammatory response in both gingival keratinocytes and dendritic cells. Inactive RgpA is bound to the cell surface of gingival keratinocytes in the region of lipid rafts, and using affinity chromatography, we identified RgpA-interacting proteins, including epidermal growth factor receptor (EGFR). Next, we showed that EGFR interaction with inactive RgpA stimulated the expression of inflammatory cytokines. The response was mediated via the EGFR–phosphatidylinositol 3-kinase (PI3K)-protein kinase B (AKT) signaling pathway, which when activated in the gingival tissue rich in dendritic cells in the proximity of the alveolar bone, may significantly contribute to bone resorption and the progress of PD. Taken together, these findings broaden our understanding of the biological role of gingipains, which in acting as proinflammatory factors in the gingival tissue, create a favorable milieu for the growth of inflammophilic pathobionts.

## INTRODUCTION

The development of periodontal disease (PD) is associated with a shift in the composition of bacteria in the subgingival biofilm toward pathogenic Gram-negative species (1). Chronic periodontitis, the most prevalent form of the disease, is manifested by sustained inflammation of the periodontium, resulting in resorption of tooth-supporting tissues, including the alveolar bone. Porphyromonas gingivalis is considered a major pathogen involved in the development and progression of chronic periodontitis (2). The pathogenic potential of P. gingivalis depends on gingipains (RgpA, RgpB, and Kgp), which are proteinases secreted to the bacterial surface by a type IX secretion system (T9SS) (3, 4) and released into the environment as soluble proteins or as cargo on outer membrane vesicles (OMVs) (5, 6). On mucosal surfaces, proteolytically active gingipains target antibacterial peptides, components of the complement system, antibodies, cytokines, and cell surface proteins. The limited proteolysis of protease-activated receptors (PARs) activates proinflammatory responses in platelets, gingival epithelial cells, fibroblasts, neutrophils, and osteoblasts (7–10) Conversely, gingipains taken up by cells efficiently inactivate intracellular key molecules in signaling pathways (11). Together, this leads to paralysis of the antibacterial cellular and humoral responses of the host. At the same time, the hijacking of otherwise tightly regulated pathways triggers chronic inflammation, in which P. gingivalis and other inflammophilic pathobionts proliferate. This results in the buildup of a dysbiotic biofilm on the tooth surface, below the gum line, in which periodontal pathogens thrive under the anaerobic conditions found in periodontal pockets.

The role of gingipains in chronic inflammation in the P. gingivalis-infected periodontium is well established (12). However, gingipains, as cysteine proteases, are inherently susceptible to oxidative inactivation (13), and this suggests that their range of action as proteolytically active enzymes is limited to periodontal pockets and the mucosal layer of the pocket epithelium. After crossing these barriers (14, 15), as soluble proteins or on OMVs, gingipains are exposed to an oxidative environment and encounter reactive oxygen species released by phagocytes. It is highly likely, therefore, that gingipains found deep in periodontal tissues are inactive (15). Nevertheless, inactive Rgp and Kgp can still exert potentially pathogenic biological activity as they can induce the expression of inflammatory cytokines (interleukin-1β [IL-1β] and granulocyte-macrophage colony-stimulating factor) in differentiated macrophages (16). Despite the pathological importance of such proteolytic activity-independent stimulation of inflammation occurring in the proximity of the alveolar bone, the mechanism underlying this phenomenon has not yet been examined.

In this study, we undertook a detailed investigation of the signaling pathway triggered by proteolytically inactive RgpA and identified the epidermal growth factor receptor (EGFR) as crucial in recognizing this form of the gingipain. Moreover, we revealed the role of gingipain-mediated activation of the EGFR signaling pathway in shaping the inflammatory response of cells crucial for the homeostasis of the periodontium.

## RESULTS

### The proinflammatory properties of gingipains depend on their enzymatic activity.

**FIG 1:**
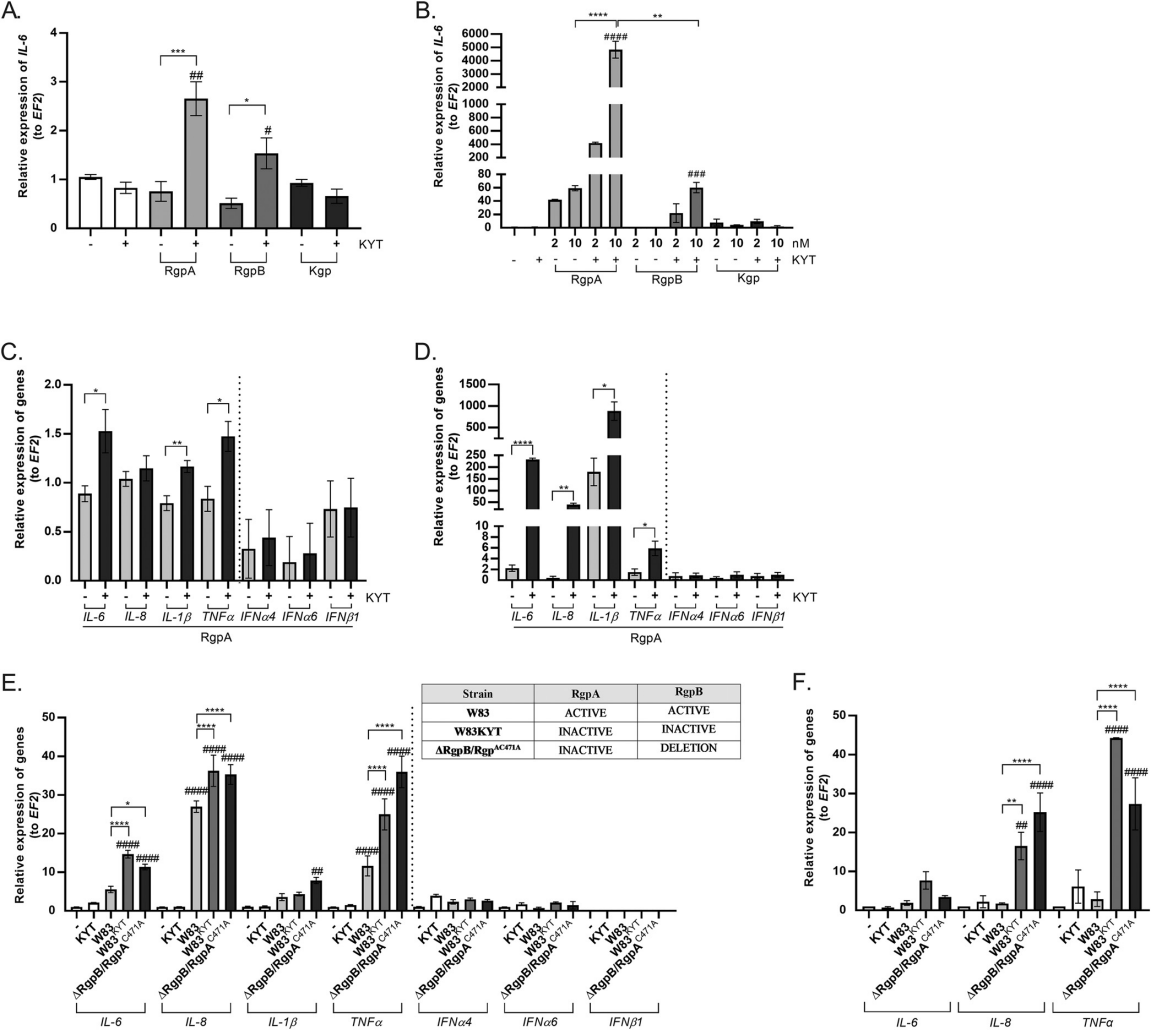
Gingipain activity determines expression of cytokines in TIGK cells. (A) Gingival keratinocytes (TIGKs) and (B) moDCs were stimulated with gingipains RgpA, RgpB, and Kgp (TIGK cells, 2 nM; moDCs, 2 and 10 nM) without or with KYT inhibitors (KYT-1 and KYT-36, each at 1 μM). Six hours after stimulation, culture media were collected, and cells were lysed with TRI reagent. Relative expression of the *IL-6* gene to the reference housekeeping gene *EF2* was measured by real-time PCR. The expression of mRNA for proinflammatory cytokine genes *IL-6*, *IL-8*, *IL-1β*, *TNF-α*, *INFα4*, *IFNα6*, and *IFNβ1* was evaluated after treatment of (C) TIGK cells or (D) moDCs with 2 nM active or inactivated RgpA. (E) TIGK cells or (F) moDCs were infected with P. gingivalis W83 in the presence or absence of specific protease inhibitors (KYT-1 and KYT-36, each at 1 μM) or an isogenic mutant expressing catalytically inactive RgpA in an *rgpB* null background (ΔRgpB/RgpA^C471A^) at an MOI of 1:25 for 6 h, and expression of mRNA for proinflammatory cytokines was analyzed. Data are presented as means ± SEM from three independent assays and are fold increase in expression compared to control levels, which were arbitrarily set at 1. *P* values are noted as follows: *#*, *P* < 0.05, #*#*, *P* < 0.01, ##*#*, *P* < 0.001, and ###*#*, *P* < 0.0001, versus control; *, *P* < 0.05, **, *P* < 0.01, ***, *P* < 0.001, and ****, *P* < 0.0001, versus P. gingivalis-infected or RgpA-treated cells.

Gingipains are sensitive to oxidative inactivation, and their diffusion into the oxygen-rich environment in gingival tissues, distant from the anaerobic milieu of the periodontal pockets, leads to loss of activity (13). This prompted us to determine the contribution of proteolytically inactive gingipains to the proinflammatory response, which is essential for inflammophilic pathogens such as P. gingivalis to thrive (17). For that purpose, we used an *in vitro* model of human telomerase-immortalized gingival keratinocytes (TIGKs). Cells were stimulated for 6 h with gingipains (RgpA, RgpB, and Kgp) in the presence or absence of KYTs, specific inhibitors of these enzymes. At the concentrations used, neither KYT inhibitors nor gingipains had an effect on the morphology or viability of TIGK cells (see Fig. S1 in the supplemental material). Initially, we focused our studies on interleukin-6 (IL-6) expression as this cytokine is crucial for the development of chronic inflammation in gingivae (18, 19). Quantitative PCR (qPCR) analysis revealed that, in contrast to the proteolytically active enzyme, the inactive forms of the arginine-specific gingipain, RgpA and RgpB (but not Kgp), are potent inducers of *IL-6* gene expression (Fig. 1A). The same effect was observed using monocyte-derived dendritic cells (moDCs), except that the response to gingipain treatment was more vigorous than that seen with TIGKs (Fig. 1B). Interestingly, in this context, inactive RgpA was a 20- and 100-fold stronger stimulator of *IL-6* expression than the inactive RgpB at concentrations of 2 and 10 nM, respectively. In addition, the amounts of IL-6 released into moDC-conditioned medium after treatment with active and inactive gingipains strongly correlated with these increases in *IL-6* mRNA (Fig. S2).

10.1128/mbio.03787-21.1 FIG S1 Gingipain RgpA used is not toxic to TIGK cells under the experimental conditions of this study. Gingival keratinocytes were stimulated with 2 nM active or inactivated RgpA, RgpB, or Kgp for 6 h. Cells were washed with PBS and stained using the ReadyProbes cell viability imaging kit, prepared according to the manufacturer’s instructions, for 15 min. After this time, dye was replaced with fresh medium, and cells were imaged using the EVOS Imaging System (60× magnification). Live cells are stained blue, and dead cells are stained green. The scale bar represents 400 μm. Download FIG S1, PDF file, 0.3 MB. Copyright © 2022 Ciaston et al. 2022 Ciaston et al. https://creativecommons.org/licenses/by/4.0/ This content is distributed under the terms of the Creative Commons Attribution 4.0 International license.

10.1128/mbio.03787-21.2 FIG S2 Gingipain activity determines expression of cytokines in moDCs and TIGK cells. Monocyte-derived dendritic cells (moDCs) were untreated or stimulated for 6 h with purified gingipains RgpA, RgpB, and Kgp (2 nM, 10 nM) in the presence or absence of specific protease inhibitors (KYT-1 and KYT-36, each at a concentration of 1 μM). The concentration of IL-6 cytokine in collected medium was evaluated by ELISA. Data are presented as means ± SEM from three independent assays. *P* values are noted as follows: ####, *P* < 0.0001 versus control; ***, *P < *0.001, and ****, *P < *0.0001, versus gingipain-treated cells. Download FIG S2, TIF file, 0.4 MB. Copyright © 2022 Ciaston et al. 2022 Ciaston et al. https://creativecommons.org/licenses/by/4.0/ This content is distributed under the terms of the Creative Commons Attribution 4.0 International license.

Since the catalytic domains of RgpA and RgpB are practically identical, the difference in the magnitudes of cell response to these gingipains is likely caused by the presence of hemagglutinin adhesion domains in RgpA (20, 21). Therefore, in subsequent studies, we focused on the ability of RgpA to stimulate cytokine expression, dependent either on nuclear factor kappa-light-chain-enhancer of activated B cells (NF-κB) transcription factors (encoded by *IL-6*, *IL-8*, *TNF-α* [tumor necrosis factor alpha], and *IL-1β*) or interferon (IFN) regulatory factors (IRFs) (encoded by *INFα4*, *INFα6*, and *INFβ1*), which are important for the development of inflammation. Stimulation of TIGKs or moDCs with enzymatically inactive RgpA significantly increased expression of mRNAs for those cytokines regulated by NF-κB, compared with that of the active form of gingipain (Fig. 1C and D). However, we found no upregulation of IRF-dependent cytokines in both tested cell types (Fig. 1C and D). Again, the magnitude of the response of moDCs to inactive RgpA was higher than the response elicited by TIGKs.

To confirm inactive gingipain-dependent, proinflammatory stimulation of gingival keratinocytes, TIGKs were infected with wild-type P. gingivalis W83 cells, W83 cells treated with KYT inhibitors (W83^KYT^), and a strain expressing catalytically inactive RgpA (RgpA^C471A^) in a Δ*rgpB* background (ΔRgpB/RgpA^C471A^) (Fig. 1E, inset). Analysis of cytokine gene expression revealed significant upregulation of NF-κB-dependent proinflammatory cytokines (*IL-6*, *IL-8*, *TNF-α*, *IL-1β*) in cells infected with the KYT-treated P. gingivalis (W83^KYT^) or ΔRgpB/RgpA^C471A^ strain (Fig. 1E). Quantitatively and qualitatively similar cytokine responses were observed for moDCs infected with KYT-treated P. gingivalis or ΔRgpB/RgpA^C471A^ strain (Fig. 1F). This response to infection with the ΔRgpB/RgpA^C471A^ strain corroborated the unique propensity of inactive RgpA to stimulate expression of proinflammatory cytokines, both in keratinocytes and in dendritic cells (Fig. 1E and F). This RgpA-specific stimulation was further confirmed with TIGKs exposed to OMVs isolated from the P. gingivalis strains tested (Fig. S3). Taken together, our data show that enzymatically inactive RgpA, the form of the enzyme most likely to occur in the oxidative environment of the periodontal tissue, is a potent stimulator of the proinflammatory, NF-κB transcription factor-dependent response of both gingival keratinocytes and dendritic cells.

10.1128/mbio.03787-21.3 FIG S3 Gingipain activity determines expression of cytokines in TIGK cells. (A) OMVs were isolated from bacterial strains, and keratinocytes were stimulated for 6 h with 6.25 μg/mL isolated vesicles. The expression of mRNA for cytokine genes *IL-6, IL-8, IL-1β, TNFα, IFNα4*, *IFNα6,* and *IFNβ1* after stimulation with OMVs was evaluated by real-time PCR. Data are fold increase in expression compared to control levels, which were arbitrarily set at 1. Data are presented as means ± standard deviations from three independent assays. *P* values are noted as follows: ###, *P* < 0.001, and ####, *P < *0.0001, versus control; **, *P < *0.01, and ****, *P < *0.0001, versus P. gingivalis-infected or RgpA-treated cells. (B) Arginine-specific gingipain activity in strains and OMVs was determined using l-BA*p*Na (200 μM) as a substrate. Download FIG S3, TIF file, 1.1 MB. Copyright © 2022 Ciaston et al. 2022 Ciaston et al. https://creativecommons.org/licenses/by/4.0/ This content is distributed under the terms of the Creative Commons Attribution 4.0 International license.

### Inactive RgpA interacts with cell membrane receptors.

**FIG 2:**
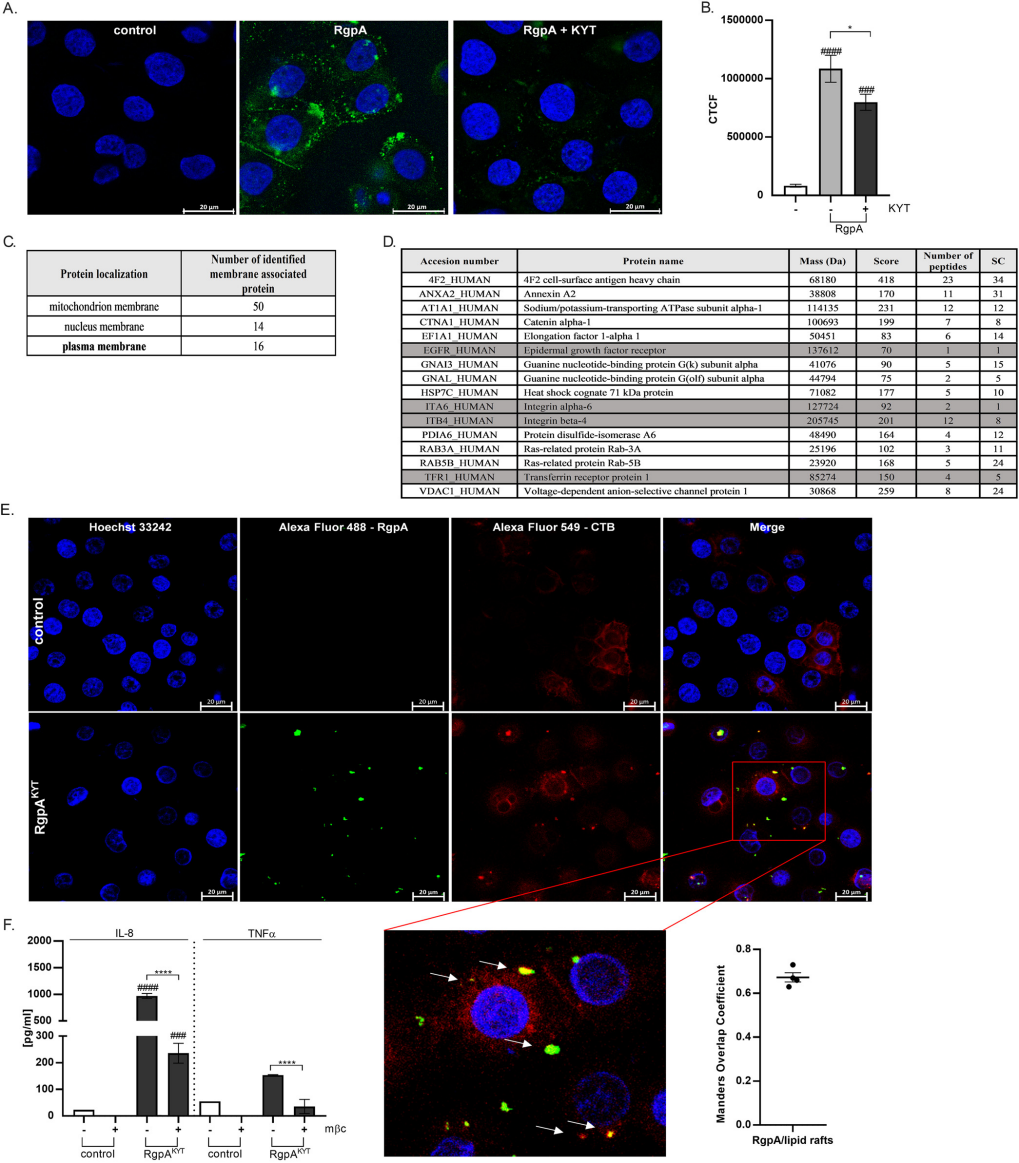
Identification of proteins on TIGK cell membrane interacting with inactive gingipain RgpA. (A) Confocal laser scanning microscopy presenting localization of RgpA (green) on TIGK cells. Cell nuclei were counterstained with Hoechst 33342 (blue). (B) Correlated total cell fluorescence (CTCF) calculated by using ImageJ software. (C, D) Membrane proteins from TIGKs that bind to inactivated gingipain RgpA. (C) Cell localization of membrane proteins identified by mass spectrometry. (D) List of potential gingipain targets detected after mass spectrometry. Only proteins localized on the plasma membrane were considered. (E) Confocal laser scanning microscopy presenting colocalization of RgpA with lipid rafts. TIGKs were stained against RgpA (green) and cholera toxin subunit B (CTB) (red). Cell nuclei were counterstained with Hoechst 33342 (blue) and analyzed at magnification of ×60. Colocalization is indicated by white arrows. The Mander’s overlap coefficient (MOC) was measured from 4 entire images. (F) Methyl-β-cyclodextrin disruption of lipid rafts in TIGKs results in a decrease of immune response induced by inactive RgpA. The concentrations of IL-8 and TNF-α were determined in supernatants of TIGKs by ELISA. Data are presented as means ± SEM from three assays. *P* values are noted as follows: ##*#*, *P* < 0.001, and ###*#*, *P* < 0.0001, versus control; *, *P* < 0.05, and ****, *P* < 0.0001, versus RgpA-treated cells.

Stimulation of the proinflammatory response in TIGKs and moDCs by inactive RgpA strongly suggests a specific interaction of the gingipain with a cell surface receptor(s). To identify this receptor, we compared binding of active or KYT-treated (RgpA^KYT^) gingipain to keratinocytes. Both forms of the enzyme attached to TIGKs in a time-dependent manner, with significant amounts of RgpA on cell surfaces after only 5 min of exposure to the gingipains (Fig. S4). Although there was no difference in the accumulation of active or inactive RgpA on TIGKs up to 30 min after exposure, significantly more of the active RgpA was detected on cell surfaces after 60 min (Fig. S2). For RgpA, the protein was present in the form of large aggregates, whereas for RgpA^KYT^, the clumps of gingipain on the cell surface were more compact (Fig. 2A and B).

10.1128/mbio.03787-21.4 FIG S4 Localization of RgpA gingipain on TIGK cells over time. Confocal laser scanning microscopy presents localization of RgpA on TIGK cells. Keratinocytes were stimulated for 0, 5, 15, 30, or 60 min with RgpA (2 nM) with or without KYT inhibitors (KYT-1, KYT-36, each at 1 μM). After fixation, slides were stained using primary antibodies against RgpA and secondary antibodies conjugated with Alexa Fluor 488 (green). Cell nuclei were counterstained with Hoechst 33342 (blue). Microscopic slides were analyzed at 60× magnification. Correlated total cell fluorescence (CTCF) from confocal microscope images was calculated using ImageJ software. Data are presented as means ± SEM from at least three independent slides. *P* values are noted as follows: #, *P <* 0.05, ##, *P* < 0.01, and ####, *P <* 0.0001 versus control. The scale bar represents 20 μm. Download FIG S4, PDF file, 0.3 MB. Copyright © 2022 Ciaston et al. 2022 Ciaston et al. https://creativecommons.org/licenses/by/4.0/ This content is distributed under the terms of the Creative Commons Attribution 4.0 International license.

Since only inactive RgpA stimulates the inflammatory response, we next attempted to identify the cell membrane molecule to which RgpA^KYT^ binds. To this end, immobilized RgpA^KYT^ was incubated with membrane proteins isolated from TIGKs, and adsorbed proteins were subjected to peptide mass spectrometry analysis. This revealed 80 membrane proteins derived from the mitochondrion (50 proteins), nucleus (14 proteins), or plasma membrane (16 proteins) (Fig. 2C). Among the plasma membrane proteins identified, there were several receptors, including EGFR, the complex of integrin alpha 6 and integrin beta 4 (integrin α4β6 complex), and the transferrin receptor (Tfr1) (Fig. 2D). These receptors are commonly localized within lipid rafts (22–24), and by applying confocal microscopy, we showed colocalization of inactive RgpA with ganglioside GM1, a marker of lipid rafts (Fig. 2E). To confirm this association, lipid rafts were disrupted using 10 mM methyl-β-cyclodextrin (mβc), and this resulted in a significant decrease in the proinflammatory response induced by RgpA^KYT^ (Fig. 2F). Cumulatively, these results strongly argue that the enzymatically inactive RgpA binds to receptors within lipid rafts on the surface of keratinocytes.

### Blocking of the EGFR signaling pathway results in a decrease in the immune response induced by inactive RgpA.

**FIG 3:**
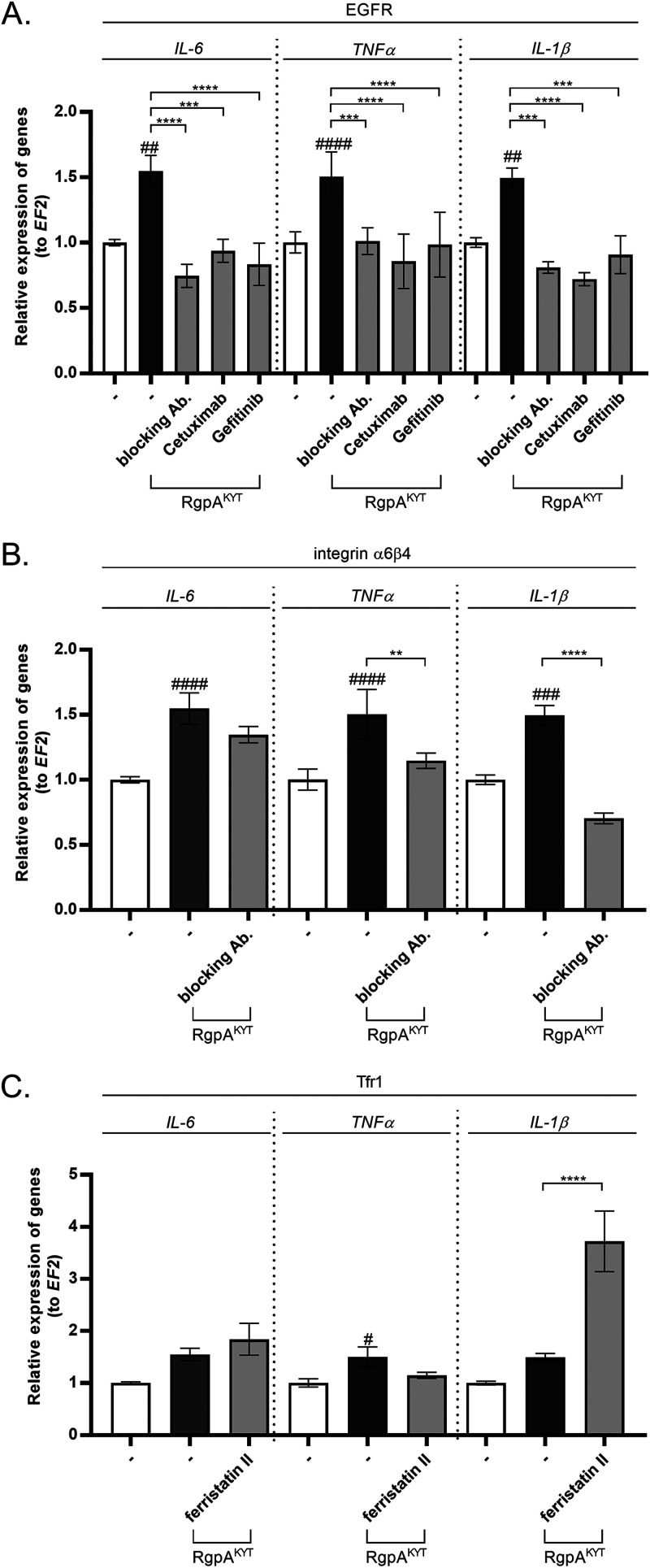
Blocking of receptors results in reduction of cytokine expression induced by inactive RgpA. TIGK cells were treated for 2 h with (A) inhibitors of the EGFR signaling pathway, including anti-EGFR neutralizing antibodies (5 μg/mL), cetuximab (10 μg/mL), and gefitinib (500 nM), (B) an inhibitor of the integrin α6β4 signaling pathway, anti-integrin β4 antibody, clone ASC-3 (10 μg/mL), and (C) an inhibitor of the Tfr1 signaling pathway, chlorazol black (ferristatin II) (20 μM). Cells were stimulated with inactive RgpA (2 nM) for 6 h, followed by evaluation of mRNA for *IL-6*, *TNF-α*, and *IL-1β* expression by real-time PCR. Data are fold increase in expression compared to control levels, which were arbitrarily set at 1. Data are presented as means ± SEM from three independent assays. *P* values are noted as follows: *#*, *P* < 0.05, #*#*, *P* < 0.01, ##*#*, *P* < 0.001, and ###*#*, *P* < 0.0001, versus control; **, *P* < 0.01, ***, *P* < 0.001, and ****, *P* < 0.0001, versus RgpA^KYT^-treated cells.

The binding studies revealed RgpA^KYT^ interaction with EGFR, integrin α6β4, and the Tfr1 receptor (Fig. 2D). To identify which of these receptors is responsible for stimulating the proinflammatory signaling pathway, TIGK receptors were selectively blocked prior to challenge of the cells with RgpA^KYT^. Baseline levels of *IL-6*, *TNF-α*, and *IL-1β* expression were observed in TIGKs pretreated with EGFR blockers, neutralizing antibody, cetuximab (25), and the tyrosine kinase inhibitor gefitinib (26) (Fig. 3A). Conversely, blocking the integrin α6β4 signaling pathway abolished the upregulation of *IL-1β* and *TNF-α* only (Fig. 3B). Blocking the Tfr1 receptor with ferristatin II slightly affected *TNF-α* expression only (Fig. 3C). Taken together, these results confirmed that the interaction of inactive RgpA with EGFR and integrin α6β4 can initiate proinflammatory signaling, with EGFR playing a major role in the response of TIGKs to treatment with RgpA^KYT^.

### Enzymatically inactive RgpA induces production of proinflammatory cytokines via the EGFR-PI3K-AKT signaling pathway.

**FIG 4:**
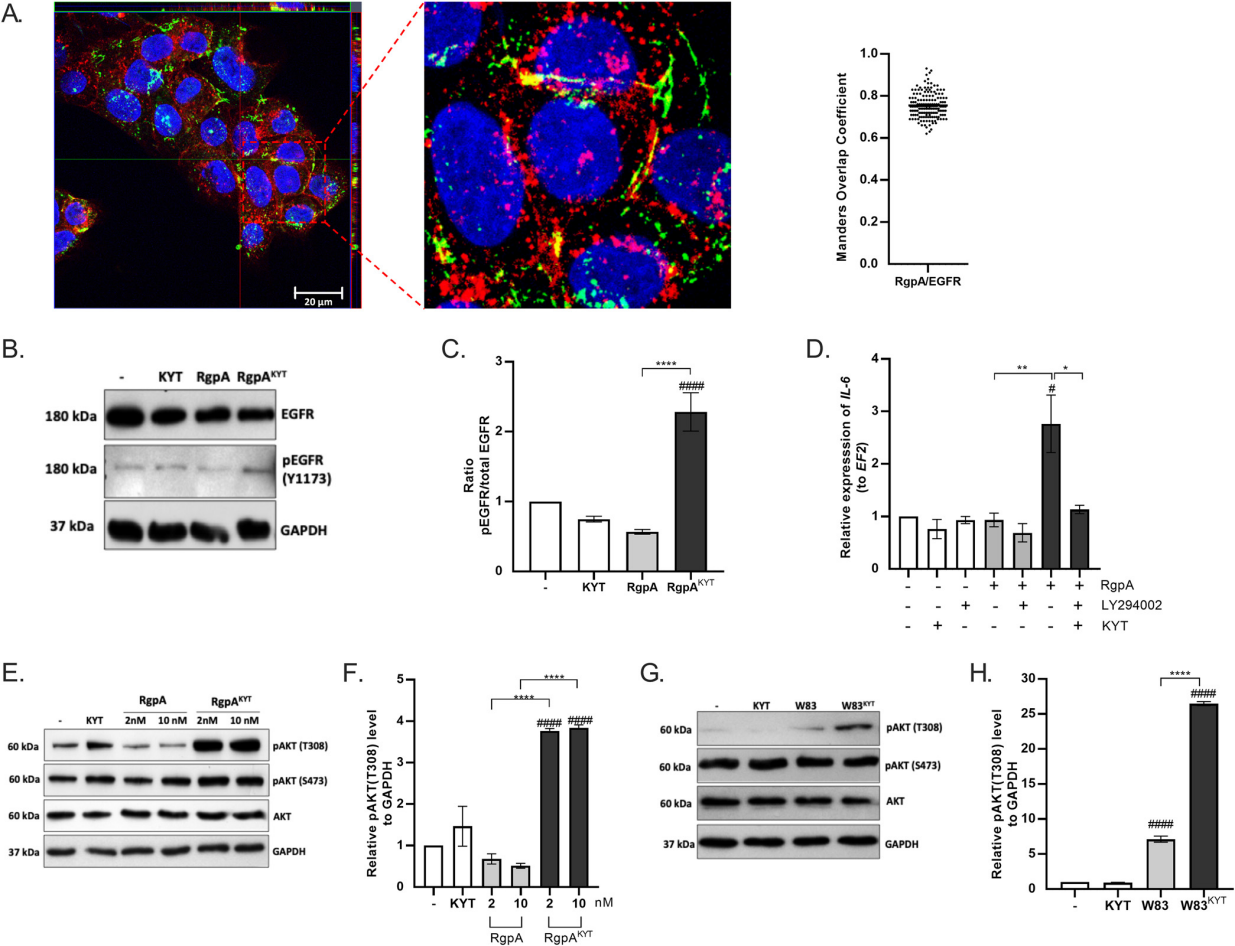
Inactive gingipain RgpA activates immune responses via the EGFR-PI3K-AKT pathway. (A) Confocal laser scanning microscopy presenting colocalization of RgpA with EGFR. Gingival keratinocytes were stimulated with inactivated RgpA (2 nM) for 1 h. Slides were stained against RgpA (Alexa-Fluor 488 [green]), EGFR (Alexa-Fluor 647 [red]), and cell nuclei (Hoechst 33342, blue), and slides were analyzed at ×100 magnification. The Mander’s overlap coefficient (MOC) was measured from 27 images. (B) Representative Western Blot analysis of phosphorylation of the EGFR receptor after 30 min of stimulation with active and inactive RgpA (2 nM). (C) Ratio of pEGFR(Y1173) to total EGFR. (D) Gingival keratinocytes were stimulated for 3 h with PI3K inhibitor LY294002 (10 μg/mL), and then active or inactivated gingipain RgpA (2 nM) was added. After 6 h of stimulation, expression of *IL-6* mRNA was evaluated by real-time PCR. (E, G) Representative Western blot analysis of phosphorylation of AKT after 1 h of stimulation with (E) active and inactivated RgpA (2 nM) or (G) strain W83 in the presence or absence of KYT inhibitors (KYT-1 and KYT-36, each at 1 μM). (F, H) Relative expression of pAKT(T308) to GAPDH. Data are presented as means ± SEM from three independent assays. *P* values are noted as follows: #, *P* < 0.05, and ###*#*, *P* < 0.0001 versus control; *, *P* < 0.05, **, *P* < 0.01, and ****, *P* < 0.0001, versus RgpA- or bacterium-treated cells.

As EGFR was identified as the primary surface molecule engaged in recognition of the inactive RgpA, we studied the signaling pathway induced by the interaction between these two molecules. First, using confocal microscopy, we confirmed this interaction by showing colocalization of gingipain and EGFR on the surface of TIGKs (Fig. 4A). The degree of colocalization was 0.75 ± 0.06, as quantified using Mander’s overlap coefficient (Fig. 4A, right panel). Next, we showed that binding of RgpA to EGFR leads to the phosphorylation of a tyrosine residue, Y1173, in the receptor molecule. Notably, phosphorylation of this residue was not observed following cell treatment with active RgpA (Fig. 4B and C). This correlates with the data in Fig. 1 showing the inability of active gingipain to induce signaling (Fig. 1). Since phosphorylated EGFR activates phosphatidylinositol 3-kinase (PI3K) and protein kinase B (AKT) (27), we investigated the effect of specifically inhibiting PI3K with the LY294002 inhibitor. This resulted in maintenance of *IL-6* expression at the baseline level following exposure to RgpA^KYT^ (Fig. 4D). This effect was confirmed using dendritic cells (Fig. S5A and B). Together, these results identify involvement of the PI3K pathway in the signal transmission induced by enzymatically inactive RgpA. This was further corroborated by demonstrating that RgpA^KYT^, but not active RgpA, induced phosphorylation of the AKT protein (4-fold higher than control treatment), especially at residue T308 (Fig. 4E and F). Interestingly, the level of pAKT(T308) decreased with active RgpA (Fig. 4E and F), again corroborating our finding that active gingipain does not stimulate proinflammatory cytokine expression (Fig. 1A). PI3K pathway activation was confirmed by infecting TIGKs with P. gingivalis. The activation of the AKT protein, measured by phosphorylation of the kinase at the T308 residue, was highest following infection of TIGKs with P. gingivalis devoid of gingipain activity (W83^KYT^) (Fig. 4G and H). P. gingivalis W83 cells treated with KYT inhibitors induced a 25-fold increase in phosphorylation, compared with around a 5-fold increase with the untreated W83 strain (Fig. 4H). Taken together, our data show, for the first time, that activation of the inflammatory signaling pathway by enzymatically inactive RgpA is mediated via EGFR recognition and activation of the PI3K-AKT signaling pathway.

10.1128/mbio.03787-21.5 FIG S5 Inhibition of the PI3K pathway reduces IL-6 production induced by inactive RgpA gingipain. Dendritic cells (moDCs) were stimulated for 3 h with the PI3K inhibitor LY294002 (10 μg/mL), and then gingipain RgpA (2 nM) was added in the presence or absence of KYT inhibitors (KYT-1 and KYT-36, each at 1 μM). After 6 h of stimulation cells were lysed in TRI reagent and (A) expression of mRNA for the *IL-6* gene was evaluated by real-time PCR. Culture media were collected and the concentration of IL-6 was evaluated (B). Data are fold increase in expression compared to control levels, which were arbitrarily set at 1. Data are presented as means ± SEM from three independent assays. *P* values are noted as follows: ###, *P* < 0.001, and ####, *P* < 0.0001, versus control; ***, *P* < 0.001, and ****, *P* < 0.0001, versus RgpA-treated cells. Download FIG S5, TIF file, 0.4 MB. Copyright © 2022 Ciaston et al. 2022 Ciaston et al. https://creativecommons.org/licenses/by/4.0/ This content is distributed under the terms of the Creative Commons Attribution 4.0 International license.

## DISCUSSION

Cell signaling, via EGFR, synchronizes the proliferation, adhesion, migration, survival, and differentiation of keratinocytes (27). In PD development, activation of EGFR slows down the progress of disease by upregulating repair of the periodontal ligament (28). Consequently, EGFR agonists are considered prospective therapeutic agents in the healing and regeneration of periodontal lesions (28). The beneficial effects of EGFR in the inflamed, P. gingivalis-infected periodontium are likely abrogated by receptor inactivation by a P. gingivalis peptidylarginine deiminase (PPAD), which efficiently blocks proliferation and migration of epidermal cells *in vitro* (29). Active gingipains can also efficiently degrade this receptor on cell surfaces (30). However, in contrast to data published by Hočevar et al. (30), we did not observe degradation of EGFR in TIGK cells. This discrepancy is probably a result of using a lower concentration of RgpA in our experiments. On the other hand, recent studies showed that inhibition of EGFR reduced the severity of bone loss and the inflammatory response in an experimental mouse ligature model of periodontitis (31). It was also demonstrated that EGFR agonists attenuated transforming growth factor β1 (TGF-β1) signaling, leading to the suppression of β6 integrin and enhancement of the proinflammatory response. Moreover, the activation of EGFR signaling occurs not only in response to expression of an autocrine EGFR ligand, but also by bacterial biofilm-derived components. For example, lipopeptide (FSL-1), which is derived from the cell membrane lipoprotein LP44 of the common oral bacterium Mycobacterium salivarium, and other bacterium-derived molecules can act as direct agonists of EGFR or via Toll-like receptor (TLR) transactivation. Together, these findings argue that permanent stimulation of EGFR may be harmful for patients with periodontitis (31).

In this study, we demonstrated that RgpA with its active site blocked by KYT-1, a specific Rgp inhibitor, is a very potent proinflammatory agonist of EGFR. The proinflammatory potency of enzymatically inactive RgpA was confirmed in an *in vitro* infection model of keratinocytes, using a genetically modified P. gingivalis strain expressing an inactive RgpA, in which the catalytic cysteine 471 residue was substituted for by alanine. We showed that P. gingivalis, either expressing inactive RgpA or treated with KYT-1, induces a significantly stronger inflammatory response than untreated, wild-type P. gingivalis. This finding corroborates previously published results showing that heat-inactivated P. gingivalis potently induces production of IL-8 (32) in fibroblasts and IL-6 (33) in human gingival epithelial cells. The lack of signal induction by active gingipains is likely due to efficient degradation of the EGFR on the cell surface (30). Together, these results suggest that P. gingivalis-induced signaling, via EGFR, depends on oxidative inactivation of gingipains, which, *in vivo*, is likely to occur in periodontal tissues distant from the tooth surface biofilm.

Gingival epithelial keratinocytes and antigen-presenting cells efficiently recognize pathogens and, in response, produce a plethora of cytokines and other mediators of inflammation. Although this routinely leads to the elimination of invading microorganisms, periodontal pathogens, many of which are inflammophilic, have developed strategies for subverting innate immunity mechanisms and thriving in the inflammatory environment. These organisms are well adapted to take advantage of chronic, inflammatory conditions, and the induction of EGFR signaling by proteolytically inactive RgpA would help to maintain such an environment in the gingival tissues, away from periodontal pockets. Thus, inactive RgpA is a novel, previously unexplored, virulence factor of P. gingivalis. Consequently, while the therapeutic use of regulators of EGFR signaling is considered, it should be noted that treatment with gingipain inhibitors may have undesirable effects.

RgpA is the gingipain that, when enzymatically inactivated, can mediate signaling via EGFR. This is attributed to the presence of a sequential motif unique for the RgpA hemagglutinin adhesion domain but absent in Kgp, as clearly illustrated in a review of the domain structure of gingipains and HagA (34). We showed that RgpA devoid of enzymatic activity strongly induced phosphorylation of tyrosine residues in EGFR, leading to activation of the PI3K-AKT pathway. This pathway is responsible for survival, proliferation, differentiation, endocytosis, metabolism, and the proinflammatory response (35, 36). Activation of AKT by inactive RgpA was accomplished by phosphorylation of T308, predominantly, suggesting the involvement of a phosphoinositide-1-dependent kinase, the PDK1 protein. This is in striking contrast to enzymatically active RgpA, which decreases phosphorylation of AKT and leads to the degradation of the native form of the kinase (37). The regulation of the AKT kinase pathway by proteolytically active gingipains is also likely to involve degradation of EGFR. In periodontitis, activation of the AKT kinase pathway by inactive RgpA may have severe pathological consequences. As mentioned earlier, gingipains, as cysteine proteases, would be subject to oxidative inactivation in tissues distant from the anaerobic environment of periodontal pockets. It is known, however, that gingipains can penetrate deeply into periodontal tissues as soluble proteins or, more likely, carried on OMVs, and they have been found distant from the pocket epithelium in biopsy specimens collected from periodontitis sites (15). Although the activity of these diffused gingipains was not determined, it is likely that they would be enzymatically inactive and, thus, capable of stimulating the AKT kinase pathway in dendritic cells, which are also located in the periodontal tissues. Since dendritic cells respond to inactive RgpA very vigorously, this would fuel local inflammation in the proximity of the alveolar bone, thus contributing to bone destruction and resorption.

Induction of the AKT pathway not only activates the inflammatory response, but also modulates the process of host cell death. Consequently, AKT activation has been postulated as a mechanism that supports the intracellular survival and growth of pathogens during infection (38). The subversion of apoptosis has been described, in detail, for many intracellular pathogens, including Shigella flexneri, which phosphorylates AKT (via IpgD protein), inhibiting apoptosis and thus promoting intracellular survival and growth of the organism (38). Interestingly, the activation of the PI3K-AKT pathway occurs also during P. gingivalis infection, and this results in host cell survival (39). P. gingivalis is a facultative intracellular pathogen and adapts to the intracellular environment (40, 41), and it is possible that activation of the AKT pathway, via enzymatically inactive gingipains, could play a role in the organism establishing a new ecological niche. The pathological role of gingipains, therefore, is not limited to the consequences of their proteolytic activity, since, as enzymatically inactive proteins, they also play a significant immunomodulatory role in cells crucial in homeostasis of the oral mucosa. Moreover, since gingipains are secreted as components of the OMV (9), they may be distributed beyond the oral cavity. Indeed, the presence of these proteins has been shown in joints, atherosclerotic plaques, and the brain. Importantly, the proteolytic activity of gingipains in those tissues was not established (42). RgpA strongly influences the biology of antigen-presenting cells, as we demonstrated for dendritic cells (DCs); thus it may play a potent role in driving T cell differentiation. Since the pathological role of enzymatically inactive gingipains is likely to extend beyond the oral cavity, they should also be considered potential contributory factors to the development of systemic diseases such as rheumatoid arthritis, atherosclerosis, and Alzheimer’s disease.

## MATERIALS AND METHODS

### Cell culture.

Telomerase-immortalized gingival keratinocytes (TIGKs) (43) were routinely cultured in KBM-Gold keratinocyte basal medium supplemented with Single Quotes (Lonza) in 37°C and 5% CO_2_. moDCs were obtained from monocytes isolated from human peripheral blood by density gradient centrifugation (44), purified using the BD human monocyte enrichment set—DM (BD IMag), and cultured as described previously (45). Blood was purchased from the Regional Blood Donation and Transfusion Center (Krakow, Poland), where materials were deidentified as appropriate to ensure human subject confidentiality. Thus, the study was excluded from human subject approval.

### Bacterial growth.

P. gingivalis W83, ΔKΔRAB (46), and ΔRgpB/RgpA^C471A^ cells were grown under anaerobic conditions (90% N_2_, 5% CO_2_, 5% H_2_) at 37°C on blood (5% [vol/vol] sheep blood) agar plates or in liquid tryptic soy broth (TSB) (30 g/L; Sigma-Aldrich) with yeast extract (5 g/L; Bioshop) supplemented with hemin (5 μg/mL; Sigma-Aldrich), l-cysteine (50 μg/mL; Sigma-Aldrich), menadione (0.5 μg/mL; Sigma-Aldrich), and in the cases of the ΔKΔRAB and ΔRgpB/RgpA^C471A^ strains, tetracycline (1 μg/mL). After overnight culture, bacteria were centrifuged (5,000 × *g*, 10 min, room temperature [RT]), and the pellet was washed three times with phosphate-buffered saline (PBS) and resuspended in PBS at a final optical density at 600 nm (OD_600_) of 1.0.

### Construction of the ΔRgpB/RgpA^C471A^ strain.

The master plasmid pNRgpA-tet, which inserts a *tetQ* antibiotic resistance cassette into the P. gingivalis genome upstream of the *rgpA* gene, was subjected to site-directed ligase-independent mutagenesis (SLIM), in which RgpA catalytic cysteine 471 was substituted for with the alanine residue (primers are listed in Table S1 in the supplemental material). The resulting plasmid (pNRgpA-C471A) was electroporated into P. gingivalis W83 ΔRgpB, and colonies of the ΔRgpB/RgpA^C471A^ strain were selected on solid medium supplemented with 1 μg/mL tetracycline.

10.1128/mbio.03787-21.7 TABLE S1 Primers used for construction of the ΔRgpB/RgpA^C471A^ strain. Download Table S1, PDF file, 0.10 MB. Copyright © 2022 Ciaston et al. 2022 Ciaston et al. https://creativecommons.org/licenses/by/4.0/ This content is distributed under the terms of the Creative Commons Attribution 4.0 International license.

### Gingipain.

Gingipains (RgpA, RgpB, Kgp) were purified from spent growth medium of P. gingivalis HG66 as described previously (47, 48). Active-site titration with specific inhibitors KYT-1 (for RgpA/B) and KYT-36 (for Kgp) (Peptide Institute, Inc.) was performed using l-BA*p*Na and *N*-(*p*-tosyl)-Gly-Pro-Lys-4-nitroanilide, respectively (Sigma-Aldrich), to determine the concertation of active gingipains (49). Substrate hydrolysis was measured at 405 nm for 40 min, and activity was presented as milli-optical density units per minute per microliter of sample. Before stimulation of eucaryotic cells, enzymes were activated with 10 mM l-cysteine as described recently (11).

### Isolation of P. gingivalis outer membrane vesicles.

Isolation of OMVs was performed as described previously (10, 11). Bacterial suspensions (OD_600_ of 1) were sonicated for 90 s in a water bath to increase the release of OMVs from the bacterial surface. Bacteria were centrifuged (10,000 × *g*, 20 min, 4°C), and collected supernatant was ultracentrifuged (150,000 × *g*, 1 h, 4°C). The OMV pellet was resuspended in buffer (20 mM Bis-Tris, 150 mM NaCl, 5 mM CaCl_2_ [pH 6.8]), and the protein concentration was evaluated by using the Pierce bicinchoninic acid (BCA) protein assay kit (Thermo Scientific).

### Stimulation of TIGKs and moDCs with P. gingivalis and its virulence factors.

TIGKs and dendritic cells (moDCs) (0.6 × 10^6^ cells) were stimulated with active gingipains RgpA, RgpB, and Kgp (2 nM, 10 nM) or inhibitor-treated forms for 30 min, 1 h, or 6 h, depending on the experiment. Eucaryotic cells were infected with the P. gingivalis W83, ΔKΔRAB, or ΔRgpB/RgpA^C471A^ mutant strain at a multiplicity of infection (MOI) of 1:25 for 1 h or 6 h. As necessary infection was performed in the presence of specific protease inhibitors KYT-1 and KYT-36, added 20 min before eukaryotic cell stimulation at a final concentration of 1 μM in 37°C. The concentration of OMVs was estimated as 6.25 μg/mL. After stimulation, culture medium was collected for enzyme-linked immunosorbent assays (ELISAs), and cells were harvested for RNA or protein isolation.

### Immunofluorescence staining.

TIGK cells (5 × 10^5^ cells) were seeded on slides and left overnight in a humid chamber (37°C, 5% CO_2_). The next day, the medium was replaced with medium containing 2 nM active or inactivated RgpA. Medium alone was used as a control. After 1 h of stimulation, cells were fixed for 10 min using 3.7% formaldehyde. Slides were washed three times with PBS and blocked for 1 h using blocking buffer (5% fetal bovine serum [FBS], 1% bovine serum albumin [BSA], 0,05% Tween 20, 2 mM EDTA in PBS) at room temperature (RT). Cells were washed once and treated with 0.1% saponin (Sigma-Aldrich) in PBS for 30 min, and then slides were incubated for 1 h with antibodies diluted in 3% BSA–0.1% saponin in PBS. For localization of RgpA, rabbit anti-RgpA antibody (10 μg/mL) was used. Colocalization of RgpA with EGFR was studied by using mouse anti-EGFR antibody (H11) (Life Technologies). Slides were washed in 0.1% saponin in PBS and incubated for 45 min with the following secondary antibodies resuspended in 3% BSA–0.1% saponin in PBS: anti-rabbit antibodies conjugated to the Alexa Fluor 488 (1:500; Life Technologies) or additionally anti-mouse secondary antibodies conjugated with Alexa Fluor 647 (1:500; Life Technologies). To visualize lipid raft structures, slides were stained for 15 min with cholera toxin subunit B (CT-B)-Alexa Fluor 594 conjugate (1 μg/mL; Molecular Probes), and for DNA identification, slides were counterstained with 1 μg/mL Hoechst 33342 (Invitrogen) for 10 min at RT. Finally, the slides were rinsed with PBS and mounted in a fluorescence mounting medium (Dako). Slides were analyzed using a Zeiss LSM 880 confocal laser scanning microscope (×60 or ×100 magnification). Correlated total cell fluorescence (CTCF) from the obtained confocal microscope images was calculated by using ImageJ software according to the equation CTCF = integrated density – (area of selected cell × mean fluorescence of background readings). Mander’s overlap coefficient was calculated from 27 independent images using ZEN black software.

### Cell viability assay.

TIGK cells (5 × 10^5^ cells) seeded on slides were stimulated with 2 nM active or inactivated gingipains for 6 h, followed by 15 min of staining using the ReadyProbes cell viability imaging kit according to the manufacturer’s instruction. Dye was replaced with fresh medium, and cells were imaged using the EVOS imaging system (×60 magnification; Thermo Fisher Scientific).

### Quantitative reverse transcription-PCR.

Total RNA was extracted from TIGKs and moDCs using TRI reagent (Sigma-Aldrich) according to the manufacturer’s instructions. cDNA was synthesized from 800 ng of RNA using a high-capacity cDNA reverse transcription kit (Applied Biosystems) according to the manufacturer’s instruction. The quantitative reverse transcription PCR (qRT-PCR) (total probe volume, 15 μL) was performed using 0.3 μL (moDCs) or 1 μL (TIGKs) of cDNA sample, 10 μM each primer, and 1× GoTaq qPCR master mix (Promega). Forward and reverse primers (Genomed) are listed in Table S2. The elongation factor 2 (*EF2*) housekeeping gene was used for normalization. After 5 min of initial denaturation (95°C) (15 min for IFNs), the reaction was carried out for 40 cycles, followed by a final elongation step at 72°C for 10 min. Conditions for (i) denaturation, (ii) annealing, and (iii) extension are listed in Table S2. Threshold cycle (*C_T_*) values were calculated using the ΔΔ*C_T_* quantification method (50).

10.1128/mbio.03787-21.8 TABLE S2 Oligonucleotides used in qRT-PCR. Download Table S2, PDF file, 0.09 MB. Copyright © 2022 Ciaston et al. 2022 Ciaston et al. https://creativecommons.org/licenses/by/4.0/ This content is distributed under the terms of the Creative Commons Attribution 4.0 International license.

### Cytokine assay.

The levels of IL-6, IL-8, and TNF-α were determined in cell supernatants by using a commercially available ELISA kit (BD Bioscience) according to the manufacturer’s instructions.

### Isolation of membrane protein from TIGK cells.

The membrane protein fraction from TIGK cells was isolated using a Mem-PER Plus membrane protein extraction kit (Life Technologies) according to the manufacturer's instructions, with some modifications. Cells were scraped from the surface of culture flasks and centrifuged at 300 × *g* for 5 min. The pellet was washed 2 times with cell wash solution and centrifuged at 300 × *g* for 5 min. Permeabilization buffer with protease inhibitor (Complete EDTA protease inhibitor; Roche) and phosphatase inhibitor (PhosSTOP; Roche) were added to the cells, and the cells were vortexed briefly and incubated for 10 min at 4°C with constant mixing. After centrifugation for 15 min at 16,000 × *g*, the cell pellet was resuspended by pipetting in solubilization buffer with protease and phosphatase inhibitors and incubated at 4°C for 30 min with constant mixing. After centrifugation at 16,000 × *g* for 15 min, the supernatant with solubilized membrane and membrane-associated protein was collected.

### Biotinylation of inhibitor-treated RgpA.

To biotinylate inactive gingipain, RgpA (RgpA^KYT^) (45 μg) was suspended in 0.1 M bicarbonate buffer (pH 8.3) and treated with biotin *N*-hydroxysuccinimide ester (1 mg in 100 μL dimethylformamide) (NHS-biotin; Sigma-Aldrich) for 4 h in 4°C with constant mixing and then dialyzed (Biomol dialysis membrane type 20; cutoff of 12 to 16 kDa and pore size of 25 Å) against PBS at 4°C for 24 h. The next day, the activity of arginine gingipain was determined, and the protein concentration was evaluated using a bicinchoninic acid (BCA) test. To maintain inhibition of RgpA, KYT inhibitors were added to samples (each at a final concentration of 1 μM).

### Affinity chromatography isolation of membrane proteins that bind gingipain.

Prebiotinylated gingipain RgpA^KYT^ was incubated at 4°C for 30 min with streptavidin-agarose gel (Merck). After washing to remove unbound gingipain, membrane proteins (in PBS with KYT inhibitors) were added and incubated at 37°C for 60 min with gentle mixing. The gel was then washed 4 times with 1 mL PBS, twice with 1 mL 300 mM NaCl, and again twice with 1 mL PBS. All washing steps were carried out in the presence of KYT inhibitors. The adsorbed gingipain-binding proteins were eluted during the gel incubation with 30 μL 2% SDS at 95°C for 20 min and separated by SDS-PAGE in the Laemmli system, with gel staining by Coomassie brilliant blue R-250. Gel control samples not combined with gingipain but incubated with membrane-derived proteins were also prepared.

### Protein identification by mass spectrometry.

Mass spectrometry was used to identify the content of the protein bands on the electrophoretic gels as described previously (51), with minor modifications. Briefly, the bands were manually excised and, after several washes in 25% and 50% acetonitrile (ACN), reduced with 100 μL of 10 mM dithiothreitol in 25 mM ammonium bicarbonate buffer (NH_4_HCO_3_) at 37°C for 45 min and then alkylated with 100 μL of 50 mM iodoacetamide in 25 mM NH_4_HCO_3_ for 1.5 h at RT in the dark. Residual reagents were removed with 200 μL of 50% acetonitrile in 25 mM NH_4_HCO_3_. Gel pieces were dehydrated in 100% ACN and dried using a SpeedVac. Gel pieces were digested with 15 μL of trypsin (0.1 μg/10 μL in 25 mM NH_4_HCO_3_) overnight at 37°C. Peptides were extracted by sonication and drying with 50 μL of 50% ACN and 0.5% formic acid. After dissolution in 35 μL of 10% ACN with 0.1% formic acid, the obtained peptides were analyzed with a HCTUltra ETDII ion-trap mass spectrometer equipped with an electrospray ionization ion source (Bruker) and coupled to an ultrahigh-performance liquid chromatograph Dionex Ultimate 3000 system (Thermo Scientific). The peptides were separated on a 100- by 2.1-mm Aeris Peptide XB-C_18_ column (particle size, 3.6 μm) (Phenomenex), with a gradient of 10 to 70% 0.1% formic acid in 80% acetonitrile in 38 min. Protein identification was performed through the Swiss-Prot protein database with taxonomy restriction to humans, using an in-house Mascot server (v.2.3.0) (Matrix Science, London, United Kingdom); the results are listed in Table S3.

10.1128/mbio.03787-21.9 TABLE S3 Full list of proteins identified by mass spectrometry. Download Table S3, PDF file, 0.2 MB. Copyright © 2022 Ciaston et al. 2022 Ciaston et al. https://creativecommons.org/licenses/by/4.0/ This content is distributed under the terms of the Creative Commons Attribution 4.0 International license.

### Disruption of lipid rafts in TIGK cells.

TIGK cells (0.3 × 10^6^ cells) were pretreated with methyl-β-cyclodextrin (mβC) (10 mM) for 30 min, washed with PBS, and stimulated with inactive RgpA (2 nM) for 6 h. Culture medium was collected for cytokine analysis.

### Blocking of EGFR, integrin α6β4, and Tfr1 signaling pathways in TIGK cells.

TIGK cells (0.3 × 10^6^ cells) were pretreated for 2 h with inhibitors of the EGFR signaling pathway, including anti-EGFR neutralizing antibodies (5 μg/mL; Sigma-Aldrich), cetuximab (10 μg/mL; InvivoGen), and gefitinib (500 nM; InvivoGen), an inhibitor of the integrin signaling pathway, anti-integrin β4 antibody (10 μg/mL; Sigma-Aldrich), and/or an inhibitor of the Tfr1 signaling pathway, chlorazol black (ferristatin II) (20 μM; Sigma-Aldrich). In the case of Tfr1, before gingipain stimulation, cells were washed 4 times with PBS and stimulated with inactive RgpA (2 nM) for 6 h, followed by cell lysis for RNA isolation.

### Blocking of PI3K signaling pathway in TIGK cells.

TIGKs and dendritic cells (moDCs) (0.3 × 10^6^ cells) were preincubated with the PI3K signaling pathway inhibitor LY294002 (Sigma-Aldrich, no. L9908) (10 μM) for 3 h, followed by 6 h of incubation with enzymatically active and KYT-inactivated RgpA (2 nM). Culture media were collected for determination of IL-6, and cells were lysed for RNA isolation.

### Protein isolation and immunoblotting.

Cell extracts were obtained using radioimmunoprecipitation assay (RIPA) buffer (0.25% Na-deoxycholate, 0.5% Nonidet P-40, 0.05% SDS, protease inhibitor cocktail, phosphatase inhibitor, and 2.5 mM EDTA in PBS). The protein concentration was evaluated using the Pierce BCA protein assay kit (Thermo Scientific), and 20 μg of proteins was separated on 10% SDS-PAGE gels and electrotransferred onto a polyvinylidene difluoride (PVDF) membrane (Merck Millipore) in transfer buffer (25 mM Tris, 0.2 M glycine, and 20% methanol). Nonspecific binding sites on membranes were blocked with 5% skim milk in TBST buffer (20 mM Tris, 0.5 M NaCl, 0.05% Tween 20 [pH 7.5]) for 1 h at RT, followed by overnight incubation at 4°C with the relevant primary antibody: rabbit anti-EGFR (1:1,000; Cell Signaling Technology), rabbit anti-pEGFR(Y1173) (1:1,000; Cell Signaling Technology), rabbit anti-AKT (1:1,000; Cell Signaling Technology), rabbit anti-pAKT(T308) (1:1,000; Cell Signaling Technology), rabbit anti-pAKT(S473) (1:1,000; Cell Signaling Technology), or rabbit anti-GAPDH (1:5,000; Cell Signaling Technology). Membranes were washed extensively in TBST buffer and incubated for 1 h at RT with horseradish peroxidase-conjugated secondary antibody, goat anti-rabbit IgG (1:5,000; Cell Signaling Technologies). Membranes were again washed extensively in TBST buffer, developed using Luminata Crescendo substrate (Merck Millipore), and exposed to Kodak medical X-ray film. The relative protein level was analyzed by using ImageLab software (Bio-Rad).

### Statistical analysis.

All experiments were performed at least in triplicate, and results were analyzed for statistical significance using Student's *t* tests or one- and two-way analyses of variance (ANOVA). All values are expressed as means ± standard errors of the means (SEM), and differences were considered significant at *P* values of <0.05. For statistical analysis, GraphPad Prism 9 (version 9.2.0) was used.

10.1128/mbio.03787-21.6 FIG S6 Raw images of immunoblots presented in Fig. 4. Download FIG S6, PDF file, 0.1 MB. Copyright © 2022 Ciaston et al. 2022 Ciaston et al. https://creativecommons.org/licenses/by/4.0/ This content is distributed under the terms of the Creative Commons Attribution 4.0 International license.
