# Supplementary figures and images for: Proteolytic Activity-Independent Activation of the Immune Response by Gingipains from Porphyromonas gingivalis
Source: mBio. 2022 May 2;13(3):e03787-21. doi: 10.1128/mbio.03787-21 (PMC9239244; doi:10.1128/mbio.03787-21)

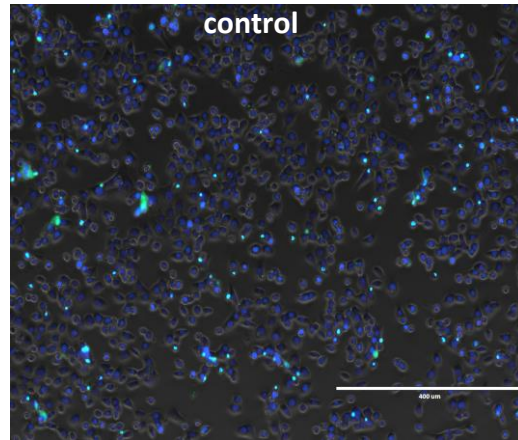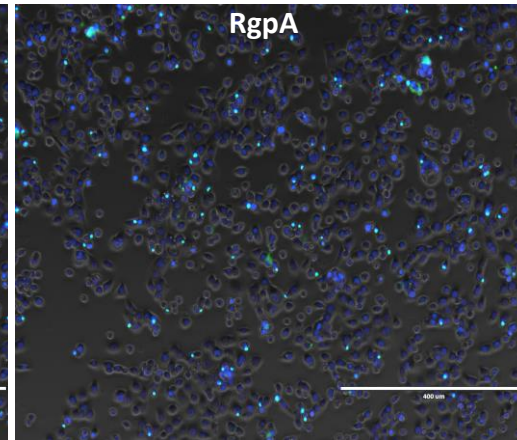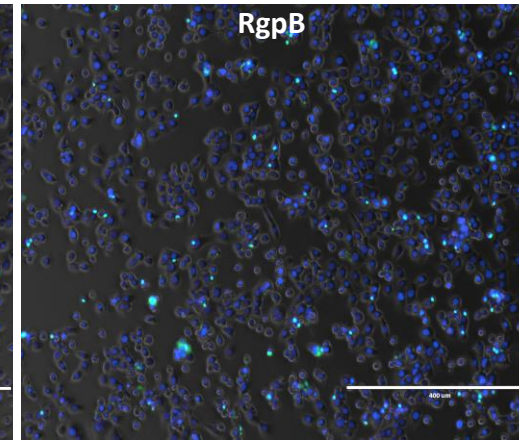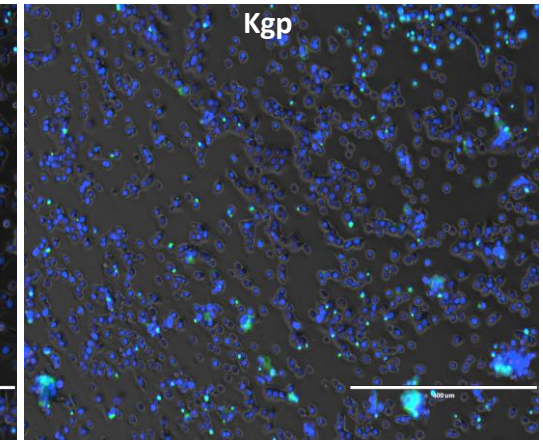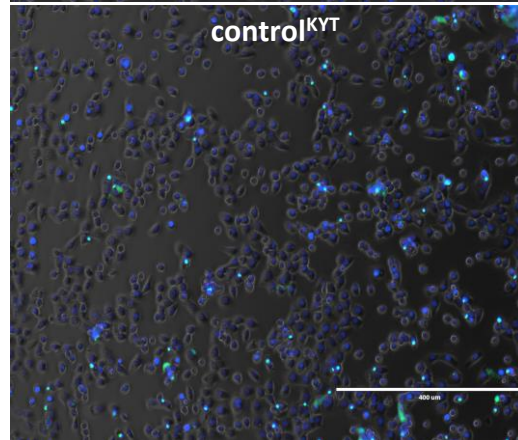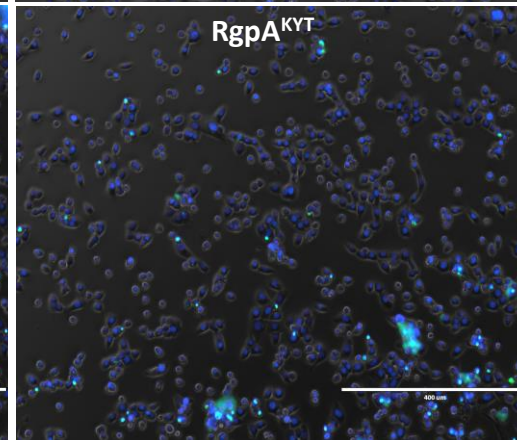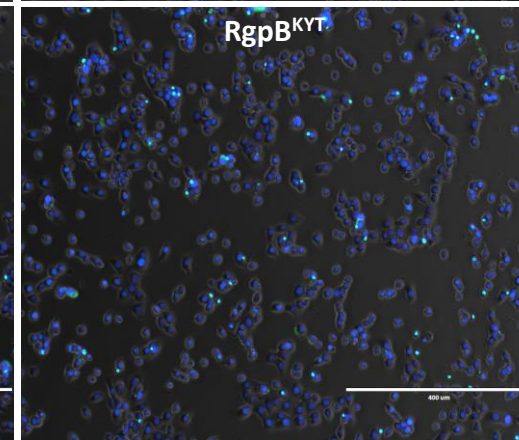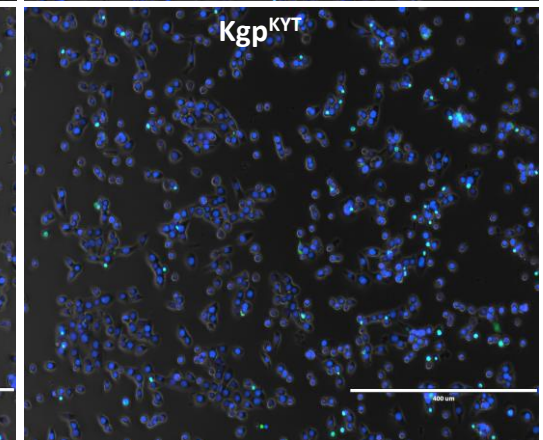

Supplement: FIG S1 [file mbio.03787-21-sf001.pdf]

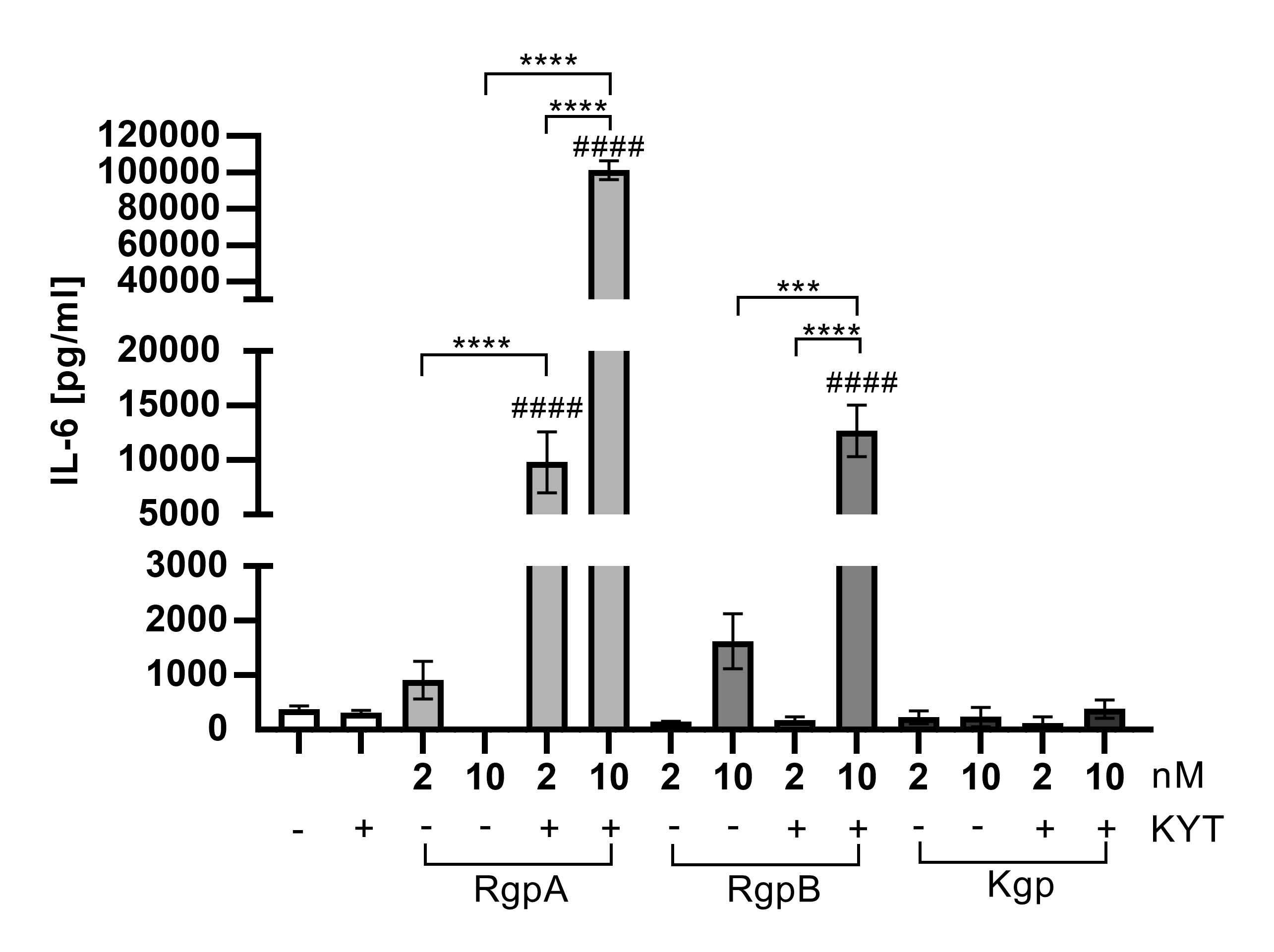

Supplement: FIG S2 [file mbio.03787-21-sf002.tif]

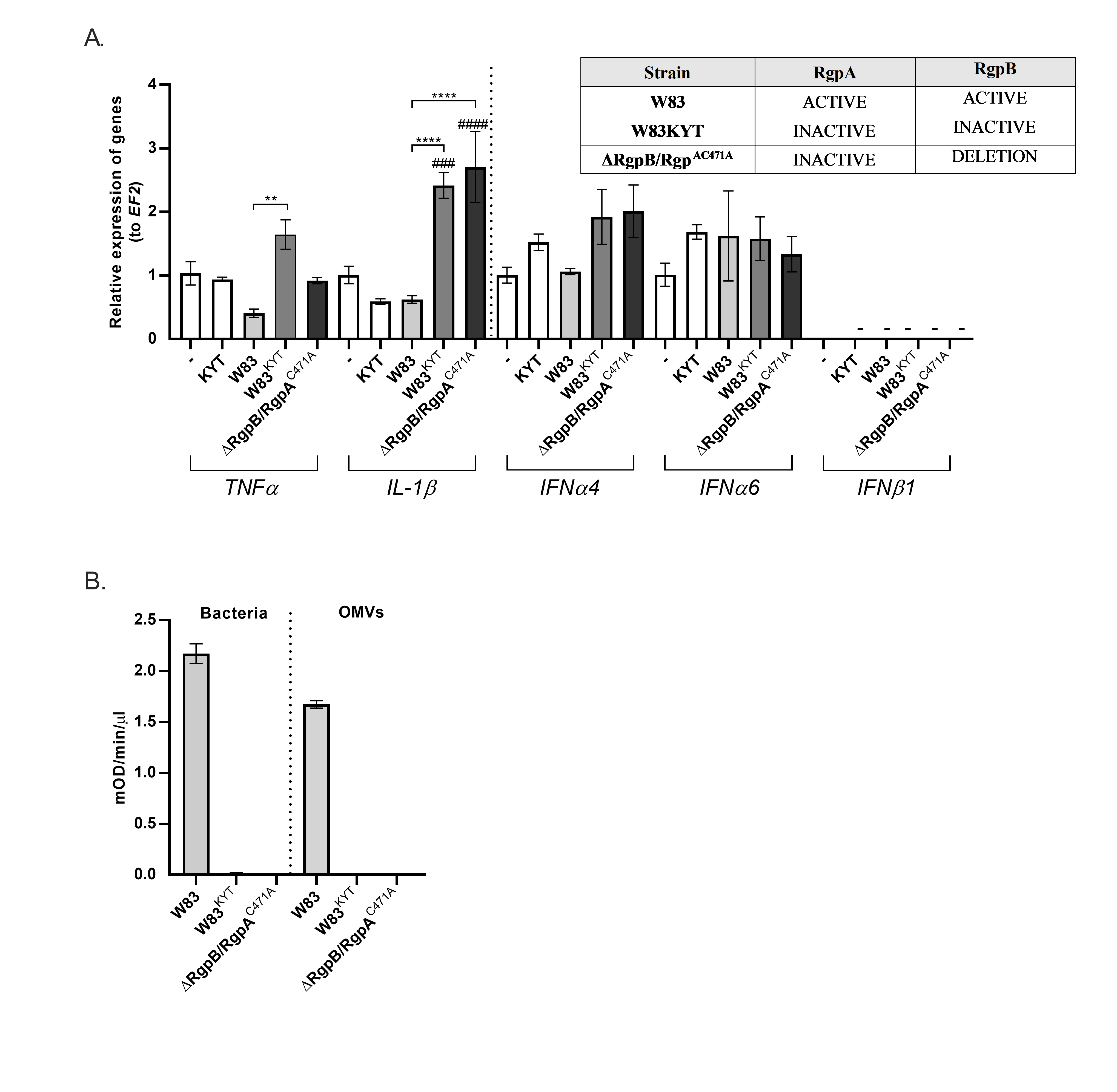

Supplement: FIG S3 [file mbio.03787-21-sf003.tif]

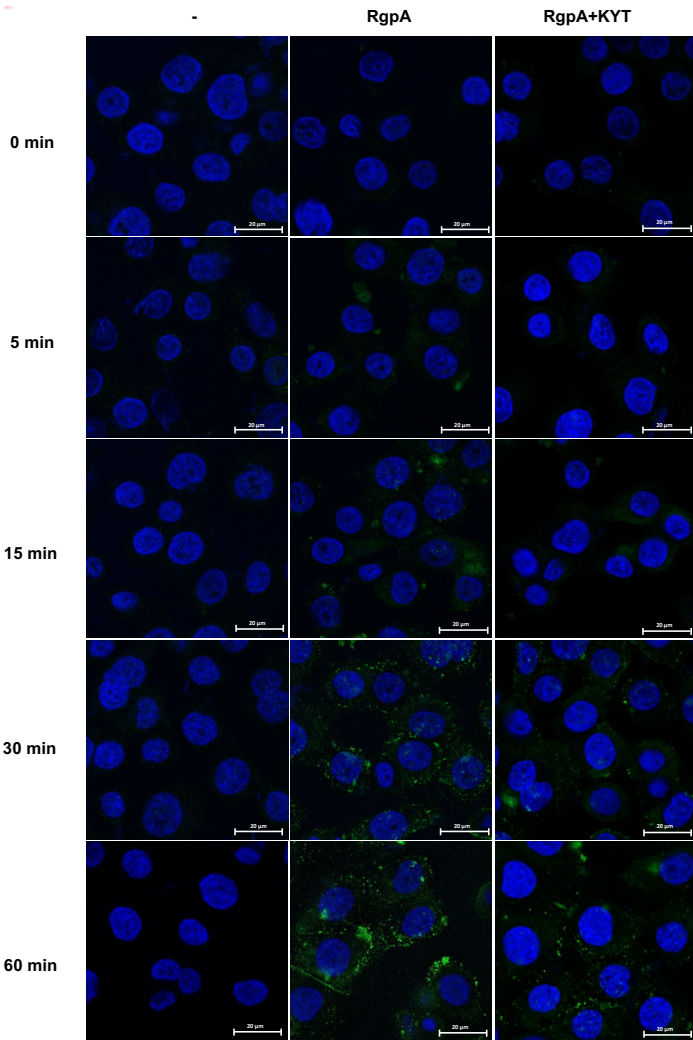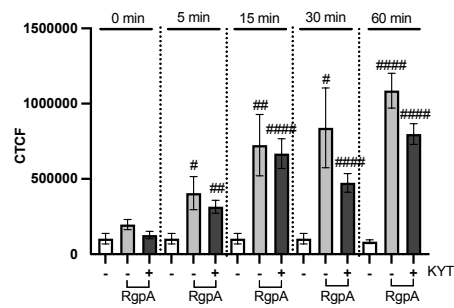

Supplement: FIG S4 [file mbio.03787-21-sf004.pdf]

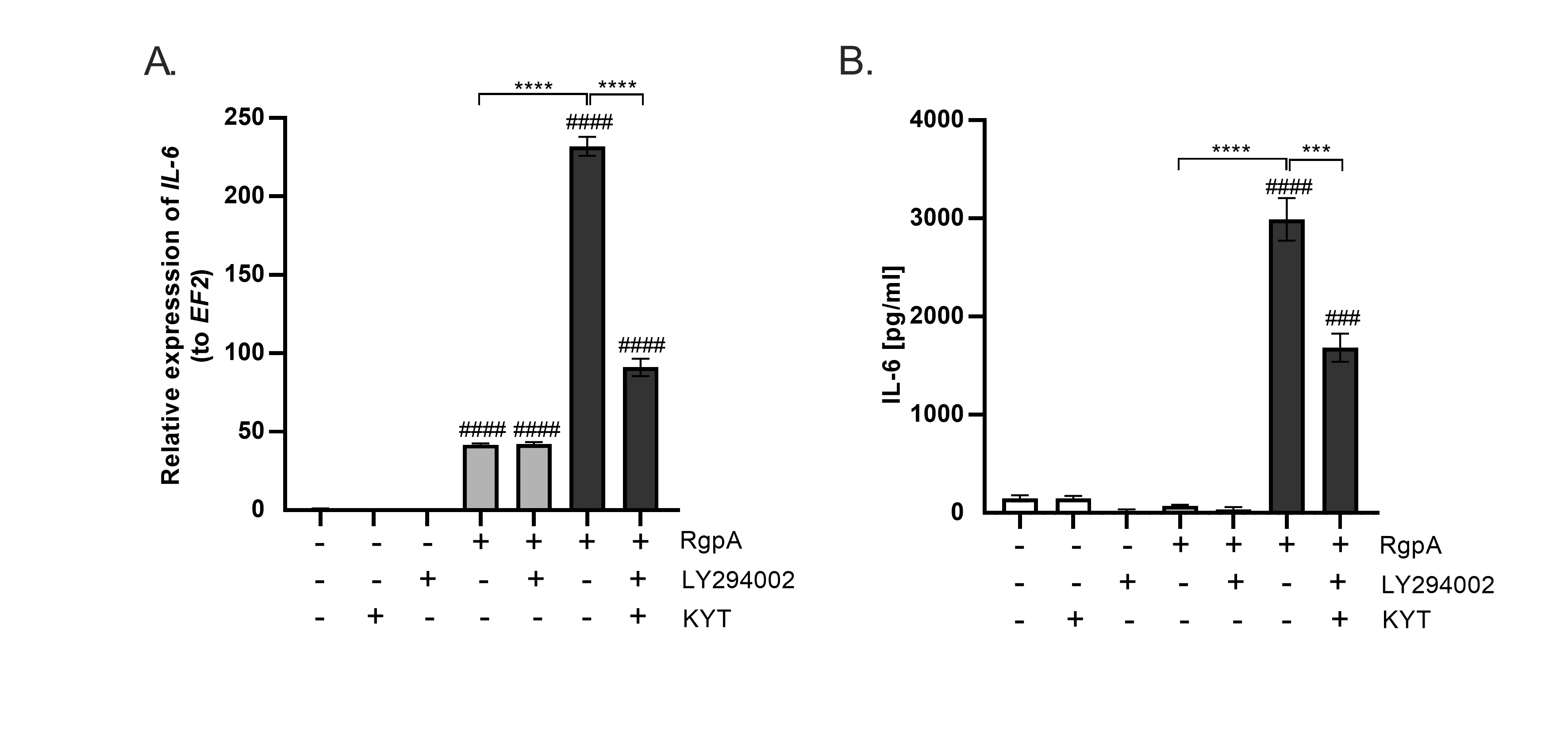

Supplement: FIG S5 [file mbio.03787-21-sf005.tif]

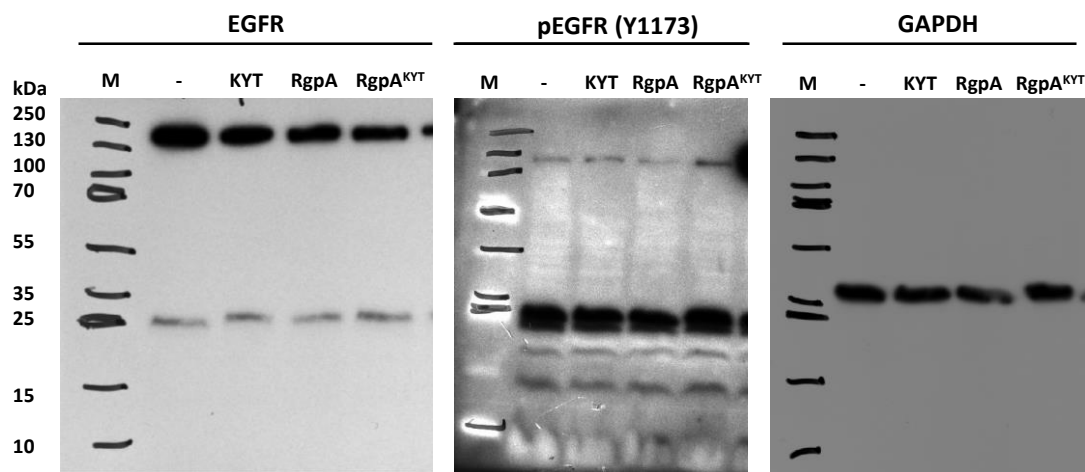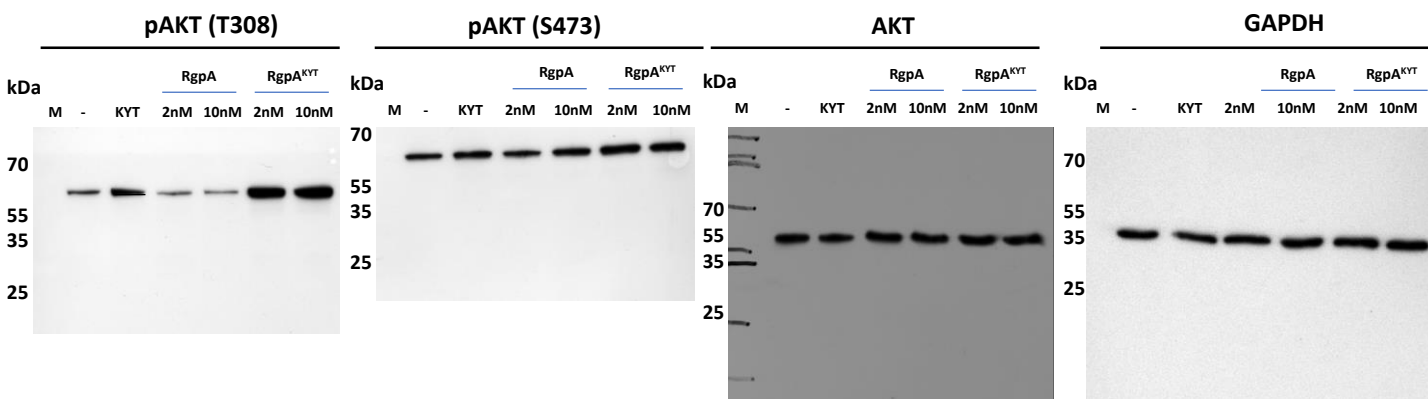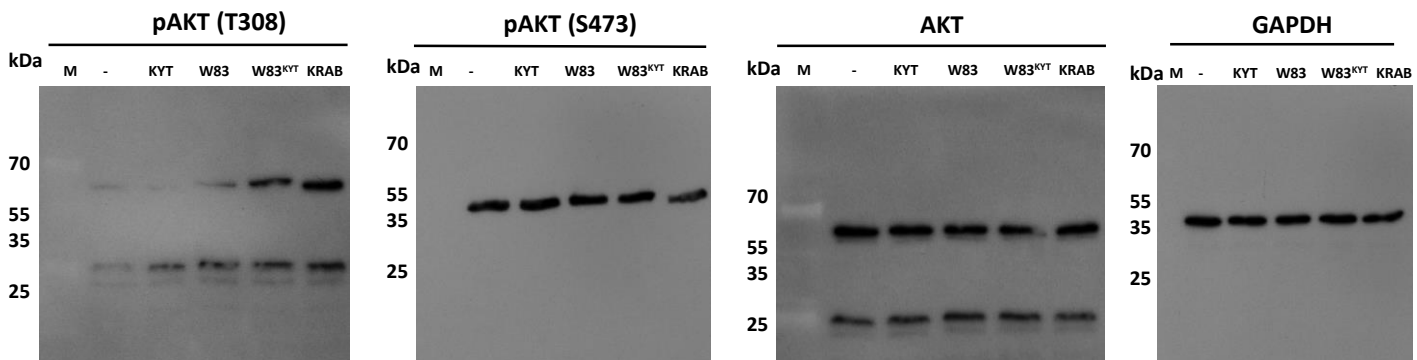

Supplement: FIG S6 [file mbio.03787-21-sf006.pdf]
