# Supplementary material for: Proteolytic Activity-Independent Activation of the Immune Response by Gingipains from Porphyromonas gingivalis
Source: mBio. 2022 May 2;13(3):e03787-21. doi: 10.1128/mbio.03787-21 (PMC9239244; doi:10.1128/mbio.03787-21)
Supplement: TABLE S1 [file mbio.03787-21-st001.pdf]

Table S1 Primers used for construction of ΔRgpB/RgpA^{C471A} strain

**pNRgpA-C471A**	
**C471A_Fs**	5’- GAATGGCGATTTCCTATTCAG – 3’
**C471A_Rs**	5’ – ATAAACGGTAGCTGGTTGCTGT – 3’
**C471A_Ft**	5’ – TTTCGACGTAGCTGCTGTGAATGGCGATTTCCTATTCAG – 3’
**C471A_RT**	5’ – ACAGCAGCTACGTCGAAAATAAACGGTAGCTGGTTGCTGT – 3’
