# Supplementary material for: Proteolytic Activity-Independent Activation of the Immune Response by Gingipains from Porphyromonas gingivalis
Source: mBio. 2022 May 2;13(3):e03787-21. doi: 10.1128/mbio.03787-21 (PMC9239244; doi:10.1128/mbio.03787-21)
Supplement: TABLE S2 [file mbio.03787-21-st002.pdf]

**Table S2 Oligonucleotides used in the qRT-PCR**

| Oligonucleotide | Sequence | Program |
|---|---|---|
| EF2 F | 5′-GACATCACCAAGGGTGTGCAG-3′ | (1)95°C,30s<br>(2)56°C,30s<br>(3)72°C,45s |
| EF2 R | 5′-TTCAGCACACTGGCATAGAGGC-3′ | |
| IL-6 F | 5′- AAATTCGGTACATCCTCGACGGCA-3′ | |
| IL-6 R | 5′- AGTGCCTCTTTGCTGCTTTCACAC-3′ | |
| TNFα F | 5′-GTCAGATCATCTTCTCGAACCCCGA-3′ | |
| TNFα R | 5′-CAGGGCAATGATCCCAAAGTAGA-3′ | |
| IL-1β F | 5′-GATGTCTGGTCCATATGAACTG-3′ | |
| IL-1β R | 5′-TTGGGATCTACACTCTCCAGC-3′ | |
| IL-23 F | 5′-GCTTTCACAGAAGCTCTGCAC-3′ | |
| IL-23 R | 5′-AGACCCTGGTGGATCCTTTG-3′ | |
| IFNα4 F | 5′-GTTCCAGAAGGCTCAAGCCATC-3′ | (1)95°C,30s<br>(2)56°C,45s<br>(3)72°C,45s |
| IFNα4 R | 5′-GTTCCAGAAGGCTCAAGCCATC-3′ | |
| IFNα6 F | 5′-GTTCCAGAAGGCTCAAGCCATC-3′ | |
| IFNα6 R | 5′-GTTCCAGAAGGCTCAAGCCATC-3′ | |
| IFNβ1 F | 5′-CTTGGATTCCTACAAAGAAGCAGC-3′ | |
| IFNβ1 F | 5′-TCCTCCTTCTGGAACTGCTGCA-3′ | |
| IL-8 F | 5′-CTCTCTTGGCAGCCTTCCTGA-3′ | (1)95°C,30s<br>(2)60°C,60s<br>(3)72°C,45s |
| IL-8 R | 5′-CCCTCTGCACCCAGTTTTCCT-3′ | |
