# Supplementary material for: Proteolytic Activity-Independent Activation of the Immune Response by Gingipains from Porphyromonas gingivalis
Source: mBio. 2022 May 2;13(3):e03787-21. doi: 10.1128/mbio.03787-21 (PMC9239244; doi:10.1128/mbio.03787-21)
Supplement: TABLE S3 [file mbio.03787-21-st003.pdf]

**Table S3. Full list of proteins identified by mass spectrometry**

| Accesion number | Protein name | Mass (Da) | Score | Number of peptides | SC |
|---|---|---|---|---|---|
| 4F2_HUMAN | 4F2 cell-surface antigen heavy chain | 68180 | 418 | 23 | 34 |
| ACAD9_HUMAN | Acyl-CoA dehydrogenase family member 9, mitochondrial | 69344 | 176 | 7 | 9 |
| ACADM_HUMAN | Medium-chain specific acyl-CoA dehydrogenase, mitochondrial | 47015 | 146 | 8 | 18 |
| ACSF2_HUMAN | Acyl-CoA synthetase family member 2, mitochondrial | 68993 | 152 | 7 | 11 |
| ACSL1_HUMAN | Long-chain-fatty-acid--CoA ligase 1 | 78919 | 136 | 9 | 11 |
| ADT2_HUMAN | ADP/ATP translocase 2 | 33059 | 174 | 10 | 31 |
| ADT3_HUMAN | ADP/ATP translocase 3 | 33073 | 83 | 5 | 16 |
| AHNK_HUMAN | Neuroblast differentiation-associated protein AHNAK | 629213 | 203 | 19 | 3 |
| AL1B1_HUMAN | Aldehyde dehydrogenase X, mitochondrial | 57626 | 138 | 3 | 6 |
| AMPL_HUMAN | Cytosol aminopeptidase | 56530 | 112 | 4 | 8 |
| ANXA2_HUMAN | Annexin A2 | 38808 | 170 | 11 | 31 |
| APMAP_HUMAN | Adipocyte plasma membrane-associated protein | 46622 | 151 | 7 | 17 |
| AT1A1_HUMAN | Sodium/potassium-transporting ATPase subunit alpha-1 | 114135 | 231 | 12 | 12 |
| AT2A2_HUMAN | Sarcoplasmic/endoplasmic reticulum calcium ATPase 2 | 116336 | 269 | 13 | 13 |
| ATPA_HUMAN | ATP synthase subunit alpha, mitochondrial | 59828 | 472 | 17 | 31 |
| ATPB_HUMAN | ATP synthase subunit beta, mitochondrial | 56525 | 682 | 24 | 43 |
| ATPG_HUMAN | ATP synthase subunit gamma, mitochondrial | 33032 | 140 | 7 | 22 |
| ATPK_HUMAN | ATP synthase subunit f, mitochondrial | 11025 | 78 | 1 | 11 |
| ATPO_HUMAN | ATP synthase subunit O, mitochondrial | 23377 | 213 | 8 | 41 |
| BACH_HUMAN | Cytosolic acyl coenzyme A thioester hydrolase | 42454 | 96 | 4 | 13 |
| CALX_HUMAN | Calnexin | 67982 | 130 | 5 | 8 |
| CH60_HUMAN | 60 kDa heat shock protein, mitochondrial | 61187 | 524 | 19 | 33 |
| CMC2_HUMAN | Calcium-binding mitochondrial carrier protein Aralar2 | 74528 | 190 | 6 | 10 |
| CTNA1_HUMAN | Catenin alpha-1 | 100693 | 199 | 7 | 8 |
| DHB4_HUMAN | Peroxisomal multifunctional enzyme type 2 | 80092 | 144 | 3 | 4 |
| ECHA_HUMAN | Trifunctional enzyme subunit alpha, mitochondrial | 83688 | 122 | 5 | 9 |
| ECHB_HUMAN | Trifunctional enzyme subunit beta, mitochondrial | 51547 | 182 | 7 | 13 |
| ECHM_HUMAN | Enoyl-CoA hydratase, mitochondrial | 31823 | 76 | 5 | 14 |
| EF1A1_HUMAN | Elongation factor 1-alpha 1 | 50451 | 83 | 6 | 14 |
| EFGM_HUMAN | Elongation factor G, mitochondrial | 84103 | 191 | 12 | 15 |
| EGFR_HUMAN | Epidermal growth factor receptor | 137612 | 70 | 1 | 1 |
| ENPL_HUMAN | Endoplasmin | 92696 | 335 | 17 | 20 |
| ETFB_HUMAN | Electron transfer flavoprotein subunit beta | 28054 | 168 | 9 | 26 |
| F213A_HUMAN | Redox-regulatory protein FAM213A | 25861 | 113 | 3 | 14 |
| FLNB_HUMAN | Filamin-B | 280157 | 177 | 11 | 5 |
| GBB1_HUMAN | Guanine nucleotide-binding protein G(I)/G(S)/G(T) subunit beta-1 | 38151 | 102 | 5 | 12 |
| GNAI3_HUMAN | Guanine nucleotide-binding protein G(k) subunit alpha | 41076 | 90 | 5 | 15 |
| GNAL_HUMAN | Guanine nucleotide-binding protein G(olf) subunit alpha | 44794 | 75 | 2 | 5 |

| | | | | | |
|---|---|---|---|---|---|
| GRP75_HUMAN | Stress-70 protein, mitochondrial | 73920 | 409 | 16 | 28 |
| GRP78_HUMAN | 78 kDa glucose-regulated protein | 72402 | 441 | 16 | 24 |
| GT251_HUMAN | Procollagen galactosyltransferase 1 | 71933 | 148 | 8 | 12 |
| HCD2_HUMAN | 3-hydroxyacyl-CoA dehydrogenase type-2 | 27134 | 99 | 3 | 12 |
| HDHD5_HUMAN | Haloacid dehalogenase-like hydrolase domain-containing 5 | 46748 | 142 | 4 | 14 |
| HNRPK_HUMAN | Heterogeneous nuclear ribonucleoprotein K | 51230 | 122 | 5 | 11 |
| HSP7C_HUMAN | Heat shock cognate 71 kDa protein | 71082 | 177 | 5 | 10 |
| HXK1_HUMAN | Hexokinase-1 | 103561 | 241 | 12 | 13 |
| HYOU1_HUMAN | Hypoxia up-regulated protein 1 | 111494 | 575 | 36 | 31 |
| IDHP_HUMAN | Isocitrate dehydrogenase [NADP], mitochondrial | 51333 | 102 | 6 | 12 |
| IPYR2_HUMAN | Inorganic pyrophosphatase 2, mitochondrial | 38409 | 127 | 7 | 25 |
| ITA6_HUMAN | Integrin alpha-6 | 127724 | 92 | 2 | 1 |
| ITB4_HUMAN | Integrin beta-4 | 205745 | 201 | 12 | 8 |
| IVD_HUMAN | Isovaleryl-CoA dehydrogenase, mitochondrial | 46803 | 121 | 3 | 8 |
| KAD4_HUMAN | Adenylate kinase 4, mitochondrial | 25366 | 103 | 5 | 26 |
| KPYM_HUMAN | Pyruvate kinase PKM | 58470 | 92 | 3 | 6 |
| LETM1_HUMAN | Mitochondrial proton/calcium exchanger protein | 83986 | 133 | 6 | 10 |
| LONM_HUMAN | Lon protease homolog, mitochondrial | 106936 | 140 | 4 | 5 |
| LPPRC_HUMAN | Leucine-rich PPR motif-containing protein, mitochondrial | 159003 | 598 | 38 | 28 |
| M2OM_HUMAN | Mitochondrial 2-oxoglutarate/malate carrier protein | 34211 | 89 | 5 | 15 |
| MCCB_HUMAN | Methylcrotonoyl-CoA carboxylase beta chain, mitochondrial | 61808 | 149 | 4 | 7 |
| MYH9_HUMAN | Myosin-9 | 227646 | 69 | 2 | 1 |
| NSDHL_HUMAN | Sterol-4-alpha-carboxylate 3-dehydrogenase, decarboxylating | 42159 | 78 | 4 | 10 |
| NU205_HUMAN | Nuclear pore complex protein Nup205 | 230171 | 139 | 6 | 2 |
| OCAD1_HUMAN | OCIA domain-containing protein 1 | 27780 | 129 | 4 | 13 |
| ODO1_HUMAN | 2-oxoglutarate dehydrogenase, mitochondrial | 117059 | 246 | 11 | 10 |
| ODO2_HUMAN | Dihydrolipoyllysine-residue succinyltransferase component of 2-oxoglutarate dehydrogenase complex, mitochondrial | 49067 | 180 | 5 | 11 |
| ODPA_HUMAN | Pyruvate dehydrogenase E1 component subunit alpha, somatic form, mitochondrial | 43952 | 186 | 4 | 11 |
| ODPB_HUMAN | Pyruvate dehydrogenase E1 component subunit beta, mitochondrial | 39550 | 166 | 7 | 22 |
| OST48_HUMAN | Dolichyl-diphosphooligosaccharide--protein glycosyltransferase 48 kDa subunit | 50940 | 176 | 4 | 9 |
| P5CR1_HUMAN | Pyrroline-5-carboxylate reductase 1, mitochondrial | 33568 | 82 | 1 | 4 |
| P5CS_HUMAN | Delta-1-pyrroline-5-carboxylate synthase | 87989 | 390 | 14 | 16 |
| PDIA3_HUMAN | Protein disulfide-isomerase A3 | 57146 | 335 | 16 | 30 |
| PDIA6_HUMAN | Protein disulfide-isomerase A6 | 48490 | 164 | 4 | 12 |
| PDPR_HUMAN | Pyruvate dehydrogenase phosphatase regulatory subunit, mitochondrial | 100156 | 210 | 8 | 10 |
| PHB_HUMAN | Prohibitin | 29843 | 168 | 8 | 30 |
| PHB2_HUMAN | Prohibitin-2 | 33276 | 324 | 15 | 50 |

| | | | | | |
|---|---|---|---|---|---|
| PRDX1_HUMAN | Peroxiredoxin-1 | 22324 | 68 | 2 | 10 |
| PRDX4_HUMAN | Peroxiredoxin-4 | 30749 | 47 | 1 | 6 |
| PTN1_HUMAN | Tyrosine-protein phosphatase non-receptor type 1 | 50505 | 338 | 10 | 22 |
| QCR1_HUMAN | Cytochrome b-c1 complex subunit 1, mitochondrial | 53297 | 72 | 1 | 2 |
| QCR2_HUMAN | Cytochrome b-c1 complex subunit 2, mitochondrial | 48584 | 112 | 7 | 14 |
| RA1L2_HUMAN | Heterogeneous nuclear ribonucleoprotein A1-like 2 | 34375 | 79 | 3 | 12 |
| RAB10_HUMAN | Ras-related protein Rab-10 | 22755 | 201 | 9 | 27 |
| RAB14_HUMAN | Ras-related protein Rab-14 | 24110 | 163 | 5 | 20 |
| RAB2A_HUMAN | Ras-related protein Rab-2A | 23702 | 174 | 4 | 21 |
| RAB3A_HUMAN | Ras-related protein Rab-3A | 25196 | 102 | 3 | 11 |
| RAB5B_HUMAN | Ras-related protein Rab-5B | 23920 | 168 | 5 | 24 |
| RAB6A_HUMAN | Ras-related protein Rab-6A | 23692 | 189 | 7 | 22 |
| RAB7A_HUMAN | Ras-related protein Rab-7a | 23760 | 299 | 11 | 50 |
| RL14_HUMAN | 60S ribosomal protein L14 | 23531 | 86 | 1 | 5 |
| RLA0_HUMAN | 60S acidic ribosomal protein P0 | 34423 | 102 | 3 | 16 |
| ROA2_HUMAN | Heterogeneous nuclear ribonucleoproteins A2/B1 | 37464 | 160 | 7 | 21 |
| RPN1_HUMAN | Dolichyl-diphosphooligosaccharide--protein glycosyltransferase subunit 1 | 68641 | 105 | 6 | 10 |
| RPN2_HUMAN | Dolichyl-diphosphooligosaccharide--protein glycosyltransferase subunit 2 | 69355 | 109 | 4 | 7 |
| RS7_HUMAN | 40S ribosomal protein S7 | 22113 | 97 | 3 | 15 |
| RT22_HUMAN | 28S ribosomal protein S22, mitochondrial | 41425 | 103 | 5 | 12 |
| RT23_HUMAN | 28S ribosomal protein S23, mitochondrial | 21814 | 90 | 4 | 22 |
| RT27_HUMAN | 28S ribosomal protein S27, mitochondrial | 47924 | 81 | 1 | 2 |
| RT29_HUMAN | 28S ribosomal protein S29, mitochondrial | 45880 | 100 | 6 | 16 |
| SDHA_HUMAN | Succinate dehydrogenase [ubiquinone] flavoprotein subunit, mitochondrial | 73672 | 151 | 8 | 16 |
| SDHB_HUMAN | Succinate dehydrogenase [ubiquinone] iron-sulfur subunit, mitochondrial | 32407 | 107 | 6 | 21 |
| SERPH_HUMAN | Serpin H1 | 46525 | 403 | 18 | 36 |
| SURF4_HUMAN | Surfeit locus protein 4 | 30602 | 74 | 2 | 7 |
| SYIM_HUMAN | Isoleucine--tRNA ligase, mitochondrial | 114688 | 239 | 16 | 17 |
| TBRG4_HUMAN | Protein TBRG4 | 71150 | 178 | 5 | 9 |
| TCPG_HUMAN | T-complex protein 1 subunit gamma | 61066 | 107 | 3 | 6 |
| TFR1_HUMAN | Transferrin receptor protein 1 | 85274 | 150 | 4 | 5 |
| THIL_HUMAN | Acetyl-CoA acetyltransferase, mitochondrial | 45456 | 143 | 9 | 21 |
| TIM50_HUMAN | Mitochondrial import inner membrane translocase subunit TIM50 | 39850 | 112 | 3 | 9 |
| TMED9_HUMAN | Transmembrane emp24 domain-containing protein 9 | 27374 | 75 | 3 | 11 |
| TMM33_HUMAN | Transmembrane protein 33 | 28302 | 66 | 1 | 4 |
| TPR_HUMAN | Nucleoprotein TPR | 267530 | 314 | 11 | 5 |
| TRAP1_HUMAN | Heat shock protein 75 kDa, mitochondrial | 80345 | 137 | 5 | 8 |
| TRI29_HUMAN | Tripartite motif-containing protein 29 | 66478 | 129 | 5 | 9 |
| TXTP_HUMAN | Tricarboxylate transport protein, mitochondrial | 34333 | 84 | 4 | 12 |
| VDAC1_HUMAN | Voltage-dependent anion-selective channel protein 1 | 30868 | 259 | 8 | 24 |

| | | | | | |
|---|---|---|---|---|---|
| VDAC2_HUMAN | Voltage-dependent anion-selective channel protein 2 | 32060 | 199 | 7 | 23 |
